# Spiraling Risk: Visualizing the multilevel factors that socially pattern HIV risk among gay, bisexual & other men who have sex with men using Complex Systems Theory

**DOI:** 10.1007/s11904-023-00664-y

**Published:** 2023-07-24

**Authors:** K. Stojanovski, E. J. King, S. O’Connell, K. S. Gallagher, K. P. Theall, A. T. Geronimus

**Affiliations:** 1 Department of Social, Behavioral and Population Sciences, Tulane School of Public Health & Tropical Medicine, New Orleans, USA; 2 Department of Health Behavior & Health Education, School of Public Health, University of Michigan, Ann Arbor, USA; 3 Department of Epidemiology, Tulane School of Public Health & Tropical Medicine, New Orleans, USA; 4 Department of Health Policy and Management, Tulane School of Public Health & Tropical Medicine, New Orleans, USA; 5 Institute for Social Research, University of Michigan, Ann Arbor, USA

**Keywords:** Systems science, HIV, Global health, GBMSM, Structural determinants, Complex systems theory

## Abstract

**Purpose of review:**

Global disparities in HIV infection, particularly among gay, bisexual, and other men who have sex with men (GBMSM), indicate the importance of exploring the multi-level processes that shape HIV’s spread. We used Complex Systems Theory and the PRISMA guidelines to conduct a systematic review of 63 global reviews to understand how HIV is socially patterned among GBMSM. The purpose was to conduct a thematic analysis of the reviews to (1) synthesize the multi-level risk factors of HIV risk, (2) categorize risk across the socioecological model, and (3) develop a conceptual model that visualizes the interrelated factors that shape GBMSMS’s HIV “risk.”

**Recent Findings:**

We included 49 studies of high and moderate quality studies. Results indicated that GBMSM’s HIV risk stems from the individual, interpersonal, and structural levels of the socioecological model. We identified a few themes that shape GBMSM’s risk of HIV infection related to biomedical prevention methods; sexual and sex-seeking behaviors; behavioral prevention methods; individual-level characteristics and syndemic infections; lived experiences and interpersonal relationships; country-level income; country-level HIV prevalence; and structural stigma. The multi-level factors, in tandem, serve to perpetuate GBMSM’s risk of HIV infection globally.

**Summary:**

The amalgamation of our thematic analyses from our systematic reviews of reviews suggests that the risk of HIV infection operates in an emergent, dynamic, and complex nature across multiple levels of the socioecological model. Applying complex systems theory indicates how multilevel factors create a dynamic and reinforcing system of HIV risk among GBMSM.

**Supplementary Information:**

The online version contains supplementary material available at 10.1007/s11904-023-00664-y.

## Introduction

Gay, bisexual, and other men who have sex with men (GBMSM) around the world experience inequitable disparities in their risk for acquiring HIV. In Europe, 50% of new HIV cases in its western region are among GBMSM [1]. In the Eastern European region, GBMSM HIV transmissions increased eight-fold from 2008–2017 [1, 2]. In 2019, in the United States, 69% of new HIV cases were among GBMSM [3] but Black GBMSM in the US account for 37% of new HIV diagnoses among GBMSM [4]. In South America, GBMSM are one of three key population groups, including transgender women and female sex workers, that account for nearly half of HIV infections [5]. In Africa, a meta-analysis indicates that 61% of GBMSM ever tested for HIV, 46% tested within the last 12 months, and only 15% of African GBMSM were engaged in care [6]. In Asia, HIV prevalence among GBMSM increased from 2007 to 2015 and ranged from 11–32% depending on the country [7]. An exploration of the factors that shape GBMSM inequities is paramount.

The literature on health behaviors and HIV among GBMSM show that current per-act probabilities of acquiring HIV, without incorporating any preventive efforts, show the highest risk stems from receptive anal intercourse at 138 infections per 10,000 exposures and, third highest from insertive anal intercourse at 11 infections per 10,000 exposures [8••]. There is upwards of a 90% reduction in HIV risk if condoms are used consistently and correctly [9]. These probabilities can be altered by pre-exposure prophylaxis (PrEP), which indicates nearly 100% protection if the regimens are adhered to appropriately [10••, 11••]. Additionally, Undetectable = Untransmittable indicates that persons living with HIV whose viral load is suppressed through adherence to treatment regimens do not transmit HIV to sexual partners [12••]. The science of HIV risk showcases numerous interrelated factors that can shape risk.

Theoretical models aim to incorporate structural and social determinants of health, which is vital for HIV science. The Health Stigma and Discrimination Framework emphasizes the social, cultural, political, and economic structures that shape stigma itself, and not merely the stigma enacted interpersonally [13]. While the framework is useful theoretically, the framework does not offer specificity in elucidating the various relationships between the social, cultural, and political levels. Rhodes et al.’s “[HIV] risk environment” shifts the focus toward the social and structural processes rather than on endogenous factors of the individual. However, the “risk environment” framework was initially conceptualized in spaces of rapid social, political, and economic transition and again didn’t elucidate the specificity of relationships [14]. The socioecological model, often presented as concentric circles, is a model that recognizes relationships between individual, interpersonal, community, organizational, and societal level factors that can all influence health, but the relationships usually are not specific [15]. Numerous studies have examined factors such as stigma, access and quality of services, and policies that can influence HIV risk. Given that multifaceted factors shape HIV risk, studying their relationships, including reinforcing and structural processes, and how the totality of factors shape HIV risk is critical. Theoretically, we can End the HIV Epidemic, yet the science of HIV is not serving all communities equitably.

Complex Systems Theory is arguably a valuable application to study HIV risk. Emergence is a key concept, which refers to the formation of the collective properties (HIV risk) arising from the systems' numerous interactions. Complex systems, as applied to HIV, recognizes that the interacting, interrelated, and dynamic processes are what create one’s HIV risk [16]. Complex systems are adaptive and dynamic in nature and have reinforcing processes that interact with one another and create dependencies and feedback loops to different parts of the system, which all, in tandem, serve to create and elevate GBMSM’S HIV risk. The emergent collective properties (HIV risk) cannot be understood by looking at system parts in isolation. Aristotle stated, “the totality is not, as it were, a mere heap, but the whole is something besides the parts…” [17]. The whole is the focus of complex systems, including how the whole gets created and emerges into existence. Complex Systems Theory has been used to visualize and specify the system of factors that shape health outcomes such as obesity, which portrays a high level of complexity of different interrelated factors across levels of the socioecological model [18]. Complex systems have not yet been applied to the body of HIV literature to identify and visualize the multi-level processes that (re)produce and socially pattern GBMSM’s HIV “risk” as a product of the system [18].

While GBMSM experience numerous risk factors for HIV that have been well studied in the literature, no single study can examine all the risk factors simultaneously. However, studying the entirety of the HIV literature will allow us to identify and visualize the multi-level factors that (re)produce HIV risk. Therefore, we used the Complex Systems Theory approach to conduct a systematic review of the HIV literature to (1) synthesize the multi-level risk factors of HIV risk, (2) thematically categorize risk across the socioecological model, and (3) develop a conceptual model that visualizes the interrelated factors that shape GBMSMS’s HIV “risk.”

## Materials and Methods

We conducted a systematic review of systematic reviews that aligned with the PRISMA criteria [19]. We worked with a librarian and research analyst to conduct the review. We used the following search terms HIV/AIDS, human immunodeficiency virus, acquired immunodeficiency syndrome; gay, bisexual, men who have sex with men, male-to-male sexual intercourse; systematic review, scoping review, meta-analysis. The full MESH search is provided in a supplementary file. We searched in CINAHL (*n* = 226), Global Health (*n* = 267), Psycinfo (*n* = 128), PubMed (*n* = 473), Scopus (*n* = 678), and Web of Science (*n* = 330) databases. We used the Covidence evidence synthesis software, which has systematized the systematic review process [20]. We identified 2,102 studies and removed 1,249 duplicates, leaving 853 systematic reviews and meta-analyses for screening (Table 1).

**Table 1:** Publication search metrics by respective database searched and removal of duplicates

Database	Number of Results (*N*)
PubMed	473
CINAHL	226
Web of Science	330
Global Health	267
Scopus	678
Psycinfo	128
# of citations (duplicates not removed)	2102
Duplicates removed in Covidence	1237
Articles to Screen	865

*Search date: 4/1/2022

**Fig. 1:**
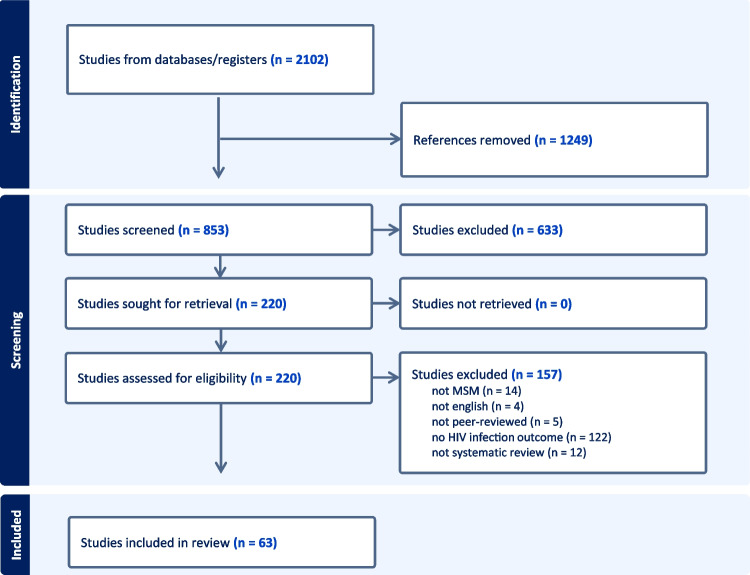
PRISMA flow diagram of the systematic review (*n* = 63)

To be included, publications were required to satisfy the following criteria: (1) had HIV infection or seropositivity as the primary outcome; (2) studied factors associated with HIV infection, whether quantitatively or qualitatively; (3) had GBMSM as a focus population; (4) were peer-reviewed systematic review or meta-analytic papers; and (6) written in English or Spanish. There was no limit on the timeframe or geographical area of the studies. Given the study is a meta-synthesis, we focused on systematic reviews & meta-analyses. Additionally, we used reviews because they interrogate discrepant information that arises in our scientific literature, ensuring high-quality data for visualization of HIV risk as a complex system. Exclusion criteria included: not having HIV infection or serostatus as the main outcome, not global in nature, no incorporation of GBMSM in study samples, non-review articles, and no examination of factors associated with HIV infection or positive serostatus. Two independent reviewers conducted the title and abstract screening (KS and SO). First, we examined titles and abstracts independently and in duplicate for inclusion and exclusion criteria. Any study that did not meet inclusion criteria after the title and abstract review was excluded (*n* = 633), leaving 220 studies for full-text review. Most studies excluded in this initial screening were either because the outcome of the studies was not HIV infection or positive serostatus or because the studies did not include GBMSM in the sample. During the full-text review, 157 papers were excluded, mainly because they did not explicitly examine factors associated with HIV infection or positive serostatus. This resulted in 63 systematic review studies for data extraction, which totaled 2,448 individual articles. Figure 1 captures the flow of title and abstract screening (reasons for exclusion are not provided at this stage in line with evidence synthesis methods) and full-text review (reasons for exclusion must be provided at this stage) from Covidence.

### Analysis and conceptualizing HIV risk

Two reviewers (KS and KG) conducted data extraction and qualitative thematic analysis of the research literature. During the full paper review, we separately extracted the following information: (1) authors; (2) year of publication; (3) geographic area of study; (4) populations under study & their frequency, and (5) identified risk factors associated with the HIV infection outcome within each review. We extracted the information and organized them into tables. We conducted quality assessments of each review using the AMSTAR-2 guidelines for systematic reviews [21]. Supplementary tables one and two present each systematic review’s strengths, weaknesses, quality assessment, and the data and results extracted from each systematic review. If multiple populations were present, we only used extracted findings specific to GBMSM. After the primary results were extracted, KS and KG categorized them into higher-order themes and respective subthemes under each level of the socioecological model (Table 2) using Bronfenbrenner’s social-ecological model. For example, condom use, the number of sexual partners, and sexual position were coded as sexual and sex-seeking behaviors (theme) under the individual level of the socioecological model. We only extracted, synthesized, and visualized data from articles with moderate or high-quality assessments (*n* = 49).

**Table 2:** Themes and subthemes identified from the systematic review of systematic reviews (*n* = 49)

Theme and subtheme	Supporting systematic review evidence (AMSTAR appraisal)
Individual-level of socioecological model	
*Biomedical prevention method shape HIV infection—1A* • Use of ART, despite condomless sex, reduces HIV risk • PrEP use reduces risk of HIV infection • Greater PrEP adherence further reduces risk of HIV infection • PEP use generally reduces risk of HIV infection • Early initiation of ARVs reduces risk of HIV infection	Bryant et al. 2009 (high) Chou et al. 2019 (high) Huang et al. 2018 (high) Jiang et al. 2020 (moderate) Okwundu et al. 2012 (high) Stromdahl et al. 2015 (high) Wang et al. 2020 (high)
*Sexual & sex-seeking behaviors influence HIV infection- 1B* • Public sex associated with higher HIV risk • Gender of sexual partners shapes HIV risk acquisition • Use of apps show mixed results for HIV risk • Transactional sex increases risk of HIV • Rectal douching increases risk of HIV infection • Condomless sex increases HIV risk • Drug use (amphetamine, party drugs, nitrites) increases risk of HIV infection • Number of sexual partners can increase HIV risk	Choi et al. 2017 (moderate) Dong et al. 2019 (high) Friedman et al. 2014 (high) Guerra et al. 2020 (moderate) He et al. 2011 (high) Hibbert et al. 2021 (high) Li et al. 2011 (high) Li et al. 2019 (high) Malta et al. 2010 (high) Oldenburg et al. 2015 (high) Vu et al. 2015 (high) Wang et al. 2015 (high) Wang et al. 2018 (moderate) Zhang et al. 2016 (high) Zhang et al. 2020 (high) Zou et al. 2017 (high)
*Behavioral prevention methods can shape HIV infection—1C* • Serosorting reduces odds of HIV infection when condoms not used • Condoms are more protective against HIV infection than serosorting • Sexual position shapes HIV risk (receptive increases risk)	Baggaley et al. 2018 (high) Baggaley et al. 2010 (moderate) Coelho et al. 2021 (moderate) Dong et al. 2019 (high) He et al. 2011 (high) Kennedy et al. 2013 (high) Li et al. 2011 (high) Malta et al. 2010 (high) Meng et al. 2015 (high) Millett et al. 2008 (high) Purcell et al. 2017 (high) Sharma et al. 2018 (moderate) Stromdahl et al. 2015 (high) Wiysonge et al. 2011 (high) Woodward et al. 2017 (moderate) Yuan et al. 2019 (high) Zhang et al. 2016 (high) Zhang et al. 2016 (high)
*Individual level characteristics & infections influence HIV infection-1D* • Lower SES associated with elevated risk of HIV infection • Higher education associated with less risk of HIV infection • Age associated with elevated risk of HIV infection • Circumcision status and HIV risk findings are mixed (protective for insertive sexual position in certain geographies) • Syphilis, Gonorrhea, Chlamydia, HPV, HBV, HSV-2 infections all associated with elevated risk of HIV infection associated with risk of HIV infection	Coelho et al. 2021 (moderate) Dong et al. 2019 (high) Freeman et al. 2006 (high) Houlihan et al. 2012 (high) Li et al. 2011 (high) Malekinejad et al. 2021 (high) Malta et al. 2010 (high) Millett et al. 2008 (high) Sharma et al. 2018 (moderate) Stromdahl et al. 2015 (high) Wiysonge et al. 2011 (high) Wu et al. 2021 (high) Yuan et al. 2019 (high) Zhang et al. 2019 (moderate) Zhang et al. 2016 (high)
Interpersonal-level of socioecological model	
*Lived experiences and interpersonal relationships shape HIV infection-2A* • Incarceration elevates HIV infection • History of childhood sexual abuse elevate risk of HIV infection • Interpersonal experiences of homophobia are associated with elevated HIV risk • Motivational interviewing as an interpersonal intervention may lower HIV risk	Berg et al. 2011 (high) Buller et al. 2014 (high) Coelho et al. 2021 (moderate) Jeffries et al. 2021 (moderate) Harawa et al. 2018 (high) LLoyd et al. 2012 (moderate) Wirtz et al. 2018 (moderate) Wiysonge et al. 2011 (high)
Structural-level of socioecological model	
*Country-level income influences HIV infection—3A* • GBMSM in low-middle income countries have lower risk of HIV than middle-income countries	Baral et al. 2007 (moderate) Blondeel et al. 2016 (high)
*Country-level prevalence shapes HIV infection- 3B* • GBMSM have higher odds of HIV infection in low prevalence countries	Baral et al. 2007 (moderate) Blondeel et al. 2016 (high)
*Structural stigma shapes HIV infection – 3C* • Country level legal protections for GBMSM reduces risk of HIV infection • Country level legal protections for sex workers reduces risk of HIV infection among GBMSM who engage in transactional sex	Oldenburg et al. 2018 (high) Stannah et al. 2018 (high)

KS used the systems mapping software, Kumu.io, to create a conceptual model [22]. The output is a conceptual model that visually represents the HIV risk environment for GBMSM, which comprises the risk factors and their potential interrelationships identified through the systematic review of systematic reviews. The risk factors identified in the review were thematically analyzed to identify the relationships between the various risk factors and HIV infection. For each risk factor (e.g., sexual behaviors), we identified relationships from the literature that conceptualized specific HIV risk pathways (e.g., no condom use < – > increased HIV risk). We used text to represent the relationships between the risk factors (e.g., decreased condom use < – > increased HIV risk). Lines portray which extracted factors are directionally associated with each other. If the studies were quantitative associational/cross-sectional, we used nondirectional arrows. We utilized unidirectional arrows when the quantitative research examined a potential causal pathway (e.g., cohort studies, intervention studies). Quantitative studies that addressed confounding were of particular interest if observational. For qualitative studies, we used major thematic outputs (i.e., family/main theme), especially for studies that had methods to achieve data saturation. In the conceptual model, we aimed to ensure the directionality of arrows was theoretically relevant and aligned with the study design (e.g., limited PrEP policies and financing would influence access and use of PrEP, rather than vice versa). The conceptual model is a visual representation of the thematic analyses that portray the complex and diverse nature that systematically pattern HIV risk among GBMSM’s. The complex systems model is a dynamic system model and thus is not meant to serve as a static visualization despite its representation within the text. If studies found negative relationships, theoretically, in a complex system, that pathway would “turn off.” The dynamic model, which respects the principles of complex systems, is available in an online supplementary file.

## Results

We included 49 systematic reviews and meta-analyses of high and moderate quality, which included a total of 1,721 studies. Among the included reviews, 35 were global (two or more continents), five only in the United States, six only in China, one in Brazil, one in Africa, and one in Europe. Thirty-two reviews explicitly focused on GBMSM, seven extended the study population beyond GBMSM to include high-risk populations such as injection drug users, sex workers, transgender people, incarcerated people, and LGBT populations more broadly. The remaining ten reviews were broader but included GBMSM in the inclusion criteria for the review. The risk of bias assessments indicated that 35 reviews were of high quality and 14 were of moderate quality. Four reviews were low quality, and nine were critically low quality (of which their data are not presented here).

The overwhelming majority (68%) of the systematic review literature on HIV infection and positive serostatus examined individual-level factors associated with infection. Eight reviews examined interpersonal-related factors, and six examined structural-level factors. Thematic analyses of the results implicated three levels of the socioecological model in the system of HIV risk: individual-, interpersonal-, and structural-level. Within the individual level, we identified (1A) biomedical prevention methods that shape HIV infection; (1B) sexual and sex-seeking behaviors influence HIV infection; (1C) behavioral prevention methods shape HIV infection; and (1D) individual-level characteristics and infections influence HIV infection as central themes. Within the interpersonal level, we identified that (2A) lived experiences and interpersonal relationships influence HIV infection. At the structural level, we found three themes: (3A) country-level income influences HIV infection; (3B) country-level HIV prevalence shapes HIV infection; and (3C) structural stigma shapes HIV infection.

### Identified themes

#### Individual-level of the socioecological model

##### Theme 1A: Biomedical prevention methods shape HIV infection

There were four reviews, one in Europe and three globally that provided high-quality information that the use of pre-exposure prophylaxis (PrEP) reduces HIV infection, emphasizing that greater adherence leads to more protection [22–24, 25••]. Wang et al. reviewed 74 studies reporting that post-exposure prophylaxis (PEP) reduces HIV risk, reporting 2.6% seroconversions were observed among 19,456 GBMSM [26••]. However, there was a lack of data from low- and middle-income countries. Two high-quality reviews, one in Europe and the other focused on the United States, Australia, and Europe, indicated that the use of, and in particular, early-initiation of ARV treatment significantly reduces the HIV infection of sexual partners [25••, 26••]. A moderate-quality global review by Jiang et al. indicated that ARV reduces HIV infection risk [27••]. Overall, the review of systematic reviews demonstrates high-quality evidence that biomedical preventative behaviors have a dramatic impact on reducing HIV infection.

##### Theme 1B: Sexual and sex-seeking behaviors influences HIV infection

Sixteen reviews discussed how sexual and sex-seeking behaviors shape HIV infection. One high-quality global review indicated that transactional sex increases the risk of HIV infection (OR 1.3, 95% CI 1.1–1.6) among GBMSM [28••]. One high-quality review in China indicated that GBMSM who had sex in bathhouses, compared to other subgroups, had the highest prevalence of HIV (OR = 13.4, 95% CI 10.3–17.1, *n* = 22) [28••]. One high-quality review in China found that GBMSM who had sex with women, as compared to men only, had an elevated prevalence of HIV (6.6% vs. 5.4% 95% CI 1.01–1.6) [29]. A review by Li 2019 found that self-reported rectal douching was associated with elevated odds of living with HIV (OR 2.8, 95% CI 2.32 to 3.39) [30••]. Several high-quality reviews and metanalyses validated the well-documented association between condomless sex and increased HIV infection [26••, 30••, 31, 31, 33••]. Four reviews six (three high and one moderate) analyzed how behavioral health factors, specifically substance use, influence HIV risk [28••, 34••, 35, 36••]. Reviews investigated a variety of drugs. Three reviews found a strong association between substance use and elevated HIV infection [34••, 35, 36••], while one in China did not find a statistically significant association [28••].

##### Theme 1C: Behavioral prevention methods can shape HIV infection

Eleven reviews examined how individual-level behaviors shape HIV infection. Four reviews (three high quality and one moderate) indicated that serosorting is less effective at HIV prevention, as compared to condoms. Still, serosorting is consistently associated with a lower risk of HIV when compared to condomless anal intercourse with no serosorting [25••, 32, 37, 38]. Eight reviews (seven high quality and one moderate quality) identified that insertive sexual positions are more protective against HIV than receptive only or receptive and insertive sexual roles [25••, 26••, 39–44]. Lastly, eight reviews (one moderate and seven high quality) consistently showed that condom use during anal intercourse prevents HIV transmission [25••, 26••, 28••, 30••, 31, 33••, 37, 45].

##### Theme 1D: Individual-level characteristics and infections influence HIV infection

Fourteen systematic reviews explored associations between HIV infection and individual-level characteristics such as socioeconomic status, education level, age, circumcision status, and syndemic infections. One moderate quality review of 47 studies from 17 countries across Latin America and the Caribbean revealed that low socioeconomic status was associated with elevated HIV prevalence [30••]. Two high-quality reviews indicated lower education levels were associated with higher HIV prevalence [28••, 33••]. Associations between age and HIV infection were mixed. Two high-quality reviews indicated that younger age was associated with more significant HIV infection [32, 34••]. In contrast, one moderate quality review showed older aged MSM had higher rates of HIV [30••]. The association between circumcision status and HIV risk has been studied extensively with mixed results. Six systematic reviews examined circumcisions and HIV, of which four reviews of three high quality and one moderate quality found no significant relationship with reduced HIV infection [25••, 40–42]. Two other high-quality reviews found some protection against HIV with male circumcision but mostly in low-middle-income countries and among insertive partners [44, 45]. The two reviews suggest that mixed results may be due to variations in study design, geography, or the country’s economic status. For example, 29 of 33 studies in Zhang et al.’s 2019 review found no significant association between circumcision and HIV infection. However, subgroup analysis revealed a protective association was found among cross-sectional studies (OR, 0.92; 95% CI, 0.87–0.98) compared to the cohort studies, which found nonsignificant associations (OR, 1.01; 95% CI, 0.86–1.19) [45]. Global data from 62 observational studies involving a total of 119, 248 MSM revealed that circumcision was protective against HIV infection among MSM in LMICs (0·58, 0·41–0·83; k = 23; I2 = 77%) but not among MSM in high-income countries (0·99, 0·90–1·09; k = 20; I2 = 40%) [44]. Lastly, HIV infection was associated with other sexually transmitted infections. Seven reviews, all high quality, indicated that the presence of other STIs, covering syphilis, gonorrhea, chlamydia, HPV, HBV, and HSV-2, was associated with higher rates of HIV infection [31, 33••, 45, 46••]. The thematic analysis of the individual-level factors reifies the complex nature of HIV risk. The body of research on individual-level factors portrays the complexity of factors that all can serve to increase risk of HIV infection.

#### Interpersonal-level of the socioecological model

##### Theme 2A: Lived experiences and interpersonal relationships influence HIV infection

Eight systematic reviews examined how an individual’s lived experience and interpersonal relationships might shape their risk of HIV. A 2018 moderate-quality review among incarcerated GBMSM from 24 middle- and high-income countries revealed that incarcerated GBMSM are at five times higher risk of HIV infection than incarcerated men who do not have sex with men [47]. However, the risk among incarcerated GBMSM varied by geography such that HIV prevalence among incarcerated GBMSM is 10 times higher in Latin America and 20 times higher in Western Europe [47]. Another high-quality review indicated that the continuity of HIV services, such as testing and testing pre-and-post release could reduce HIV cases among Black GBMSM who experience incarceration and reenter their communities post-release [48].

Three systematic reviews (two moderate and one high quality) examined the relationship between HIV infection and experiences of abuse (childhood and intimate partner violence), all indicating that GBMSM who experienced abuse had elevated HIV infections [30••, 48, 49]. A moderate 2012 systematic review of 12 papers revealed higher HIV infection among men with a history of childhood sexual abuse as compared with men with no history [OR = 1.54; 95% CI (1.22–1.95)] [50••]. Two reviews (Coelho and Buller) found positive associations between violent relationships and elevated HIV infection [30••, 48]. Buller et al.’s global review of data from 8,835 MSM found that exposure to intimate partner violence was associated with a 1.5 times greater chance of a positive HIV status [AOR = 1.5, 95% CI (1.3, 1.7)] [49].

One moderate-quality review examined how GBMSM’s experiences of interpersonal homophobia and homophobic abuse shaped HIV diagnoses. The 2021 rapid review and meta-analysis of 121 cross-sectional studies found an elevated risk of diagnosed HIV among GBMSM in the US who experienced homophobia (OR = 1.34, 95% CI = 1.10–1.64, I2 = 86.3%) [50••]. Stratified analysis showed different levels of HIV infection by race and ethnic group, with Latinos experiencing the greatest.

Lastly, a review examined motivational interviewing as an intervention to support the prevention of HIV among MSM. However, the results concluded that evidence of motivational interviewing’s effectiveness was lacking [51]. The various interpersonal-level factors that shape HIV infection serve to further complicate conceptualizations of HIV “risk.”

#### Structural-level of the socioecological model

##### Theme 3A: Country-level income influences HIV infection

Two review papers (moderate and high quality) discussed how country-level socioeconomic factors influence HIV prevalence. The moderate quality review by Baral et al. indicated a lower odds ratio for HIV infection among GBMSM in low-income countries (OR = 7.8, 95% CI 7.2, 8.4), as compared to GBMSM in middle-income countries (OR = 23.5, 95% CI 22.8, 24.0) [52••]. A review by Blondeel et al. indicated that MSM in low-middle income countries had a 19.3 increased chance of HIV infection, as compared to the general population [61*i*].

##### Theme 3B: Country-level prevalence influences HIV infection

In the same two reviews, GBMSM in very low prevalence countries had the highest OR of HIV infection [OR = 58.4, 95% CI (56.3, 60.6)], as compared to low prevalence [OR = 14.4, 95% CI (13.8, 14.9)], and high prevalence countries [OR = 9.6, 95% CI (8.9, 10.2)] [52••]. Another review showed geographic variation in HIV burden, with the highest prevalence of HIV among GBMSM found in Caribbean countries and sub-Saharan Africa, and lowest in countries within the Middle East and North Africa [52••].

##### Theme 3C: Structural stigma shapes HIV infection

Two high-quality reviews examined the role of structural stigma, including how homophobic and criminalizing policies elevate HIV infection [53••, 54••]. Oldenbrug et al.’s review indicated an 11% lower HIV prevalence in countries with protective laws for MSM, compared to countries lacking legal protections [53••]. Countries with protective language for men who sell sex had a 7% lower prevalence of HIV, as compared to those without such protections [54••]. A systematic review and meta-analysis of 75 papers found that GBMSM were more likely to be aware of their HIV status in countries with less repressive legislation (22% vs. 6.7%) and less severe penalties for same-sex relations [55]. These findings indicate that structural factors could influence HIV infection and complicate our understanding of HIV risk further.

### Dynamic and emergent nature of HIV risk

**Fig. 2:**
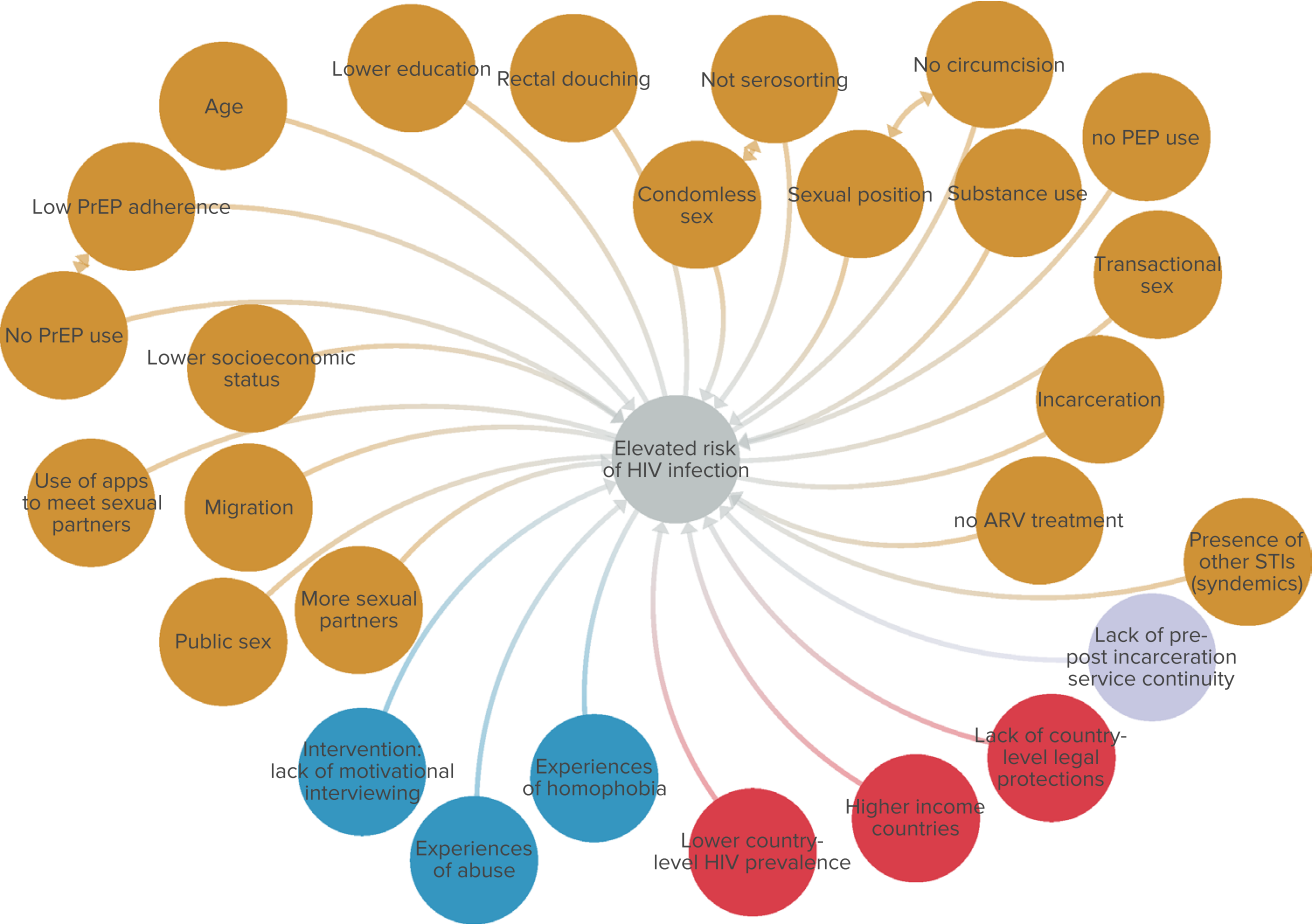
Complex systems visualization of HIV risk among GBMSM, globally. Legend: Yellow: individual-level**.** Blue: interpersonal-level**.** Green: community-level**.** Purple: institutional-level**.** Red: structural-level

In totality, the amalgamation of our thematic analyses and findings from our systematic reviews of reviews suggests that the risk of HIV infection operates in an emergent, dynamic, and complex nature across multiple levels of the socioecological model that interact with one another to elevate GBMSMS’s risk of HIV further. Structural factors such as stigmatizing policies, macroeconomic factors, and population-level epidemiology can shape environments in ways that produce HIV infection—moreover, interpersonal factors such as experiences of abuse and homophobia further shape HIV infection among GBMSM. Lastly, numerous individual-level factors such as sexual behaviors, use of biomedical prevention methods, and sexual relationships, amongst others, further perpetuate the risk of HIV infection among GBMSM. This systematic review of reviews and meta-analyses and the developed visualization indicated that HIV risk among GBMSM worldwide is socially patterned by numerous interrelated factors, such that the ecosystem itself is the driver of the disproportionate risk of HIV that burdens GBMSM (Fig. 2).

## Discussion

The results of our systematic review portray that, globally, HIV infections among GBMSM arise from a complex interplay of structural, interpersonal, and individual-level factors. The visualization positions that structural, interpersonal, and individual-level forces operate in concert to structure GBMSM’s risk of HIV infection. The totality of these relationships influences the emergence of HIV infection in ways that disproportionately burden GBMSM throughout the globe. The analyses and conceptual model suggest that focusing on the system that shapes HIV infection is needed to address the interactional and collective nature of HIV risk that GBMSM experience.

The growing application of complex systems models improves our understanding of how several intersecting processes shape population-level HIV infection and prevalence. A recent agent-based model (ABM) of a complex system explored U.S. racial inequities in PrEP use and showed that PrEP uptake will reduce overall HIV infections, it will do nothing to address the HIV disparities currently existing [56]. In the model, when Black and White agents use PrEP at equal rates, the HIV disparity ratio between the two groups continues to increase because the underlying disparity was never addressed [56]. Goedel’s findings support the need to simultaneously address the diversity of risk factors such as accessibility, acceptability, and quality of healthcare service, which align with the results of our study, portraying the system that shapes HIV infection. This aligns with a meta-analysis of 194 studies in Canada, the United States, and the United Kingdom, which showed that Black GBMSM were more likely to engage in individual-level prevention behaviors (e.g., fewer sexual partners, more condom use, less likely to use substances), as compared to White GBMSM, yet had greater odds of testing positive for HIV and six-fold greater odds of having undiagnosed HIV [57]. However, as our systematic review indicates, the scientific literature still predominately focuses on individual-level risk factors for HIV, as evidenced by the 68% of articles from 2000–2021 that focused on individual-level factors. Our findings add further credence to how HIV infection arises from a complex system of factors, not simply individual-level behaviors, and indicate that enhanced conceptualizations of HIV “risk” are needed.

This is the first study to identify and visualize the interacting processes that socially pattern the emergence of HIV risk and infection among GBMSM. Global responses, such as the Joint United Nations Programme on HIV/AIDS (UNAIDS), must adequately address the system of factors together, instead of in isolation, to achieve zero new HIV infections. Scholarship implores us to think of interventions not merely as a “package” of activities but rather, alternatively, to focus on the dynamic processes of the environment and context where interventions are introduced [58]. The focus on the dynamic processes is significant for HIV, which, as our results show, arises from multi-faceted routes across the socioecological model. Studies have also shown how country-level policies shape the ecosystem of HIV risk in ways that elevate GBMSM’s risk of HIV [59]. Research also identifies how combining multiple HIV intervention strategies may be necessary given the complexity in which HIV risk is socially patterned [60]. Further research needs to examine how risk factors are situated within the social and structural contexts to fully grapple with the multiple levels of influence that can weaken or support the capacity of GBMSM to reduce their risk of HIV infection.

Limitations in our synthesis include aspects of the approach used and generalizability. While we used a systematic review of review studies, the system of processes outlined in Fig. 2 is only partial. This is especially true as our primary outcome was HIV infection, and we didn’t include studies that might have explored risk factors for the risk factors themselves. The dynamic processes will change over time as scientific research and interventions occur. Many of the pathways developed were identified as associations, given the cross-sectional nature of much of our HIV research literature. To enhance the rigor, we removed the 22% of reviews that were low or critically low quality. Lastly, given that our data focused on reviews or meta-analyses rather than individual studies, we may be missing risk domains if no review study exists for that domain. However, focusing on reviews and meta-analyses allowed for a meta-review of our scientific literature on HIV risk to begin to grapple with the complexity of “HIV risk.”

Future research should further explore the system of HIV risk by examining what may shape the HIV risk factors (risks of risks). Automating systematic review processes using natural language processing (NLP) and machine learning could enhance the synthesis of the research literature. NLP is a collection of algorithms that can be used to identify, extract, parse, and analyze textual data, such as written text in journal articles, which is growing with tools such as GPT, ElicitAI, amongst others [61]. The application of Complex Systems Theory and our conceptual model can be leveraged to better hypothesize and study the mechanisms that shape population-level HIV inequities among priority populations across different environments, geographies, and interventions.

## Conclusion

The thematic analysis and visualization indicate that HIV infection among GBMSM, globally, is socially patterned by a diversity of numerous interacting risk factors. Applying complex systems theory indicates how multilevel factors create a dynamic and reinforcing system of HIV risk among GBMSM. Having this more complete understanding of what shapes HIV risk is critical for developing more effective HIV prevention efforts for highly burdened populations and for strengthening the global commitment to achieve zero new HIV infections.

## Supplementary Information

Below is the link to the electronic supplementary material.

Supplementary file1 (DOCX 20 KB)

Supplementary file2 (DOCX 73 KB)

Supplementary file3 (DOCX 72 KB)

## Data Availability

Data are available as tables in supplementary files.
