# Supplementary material for: Spiraling Risk: Visualizing the multilevel factors that socially pattern HIV risk among gay, bisexual & other men who have sex with men using Complex Systems Theory
Source: Curr HIV/AIDS Rep. 2023 Jul 24;20(4):206–17. doi: 10.1007/s11904-023-00664-y (PMC10403445; doi:10.1007/s11904-023-00664-y)
Supplement: Supplementary file 1 — Supplementary file1 (DOCX 20 KB) [file 11904_2023_664_MOESM1_ESM.docx]

Supplementary table 1: Search terms and logic by respective information database searched, April 2022

**PubMed**

| Search | Query | # of Results |
| --- | --- | --- |
| 1 | MSM[tiab] OR gay[tiab] OR homosexual*[tiab] OR "homosexuality"[Mesh:noexp] OR "homosexuality, male"[Mesh] OR men who have sex with men[tiab] OR bisexual*[tiab] OR "bisexuality"[mesh] | 50,055 |
| 2 | ((systematic* [ti] AND review [ti]) OR Systematic overview* [ti] OR Cochrane review* [ti] OR systemic review* [ti] OR scoping review [ti] OR scoping literature review [ti] OR mapping review [ti] OR Umbrella review* [ti] OR (review of reviews [ti] OR overview of reviews [ti]) OR meta-review [ti] OR (integrative review [ti] OR integrated review [ti] OR integrative overview [ti] OR meta-synthesis [ti] OR metasynthesis [ti] OR quantitative review [ti] OR quantitative synthesis [ti] OR research synthesis [ti] OR meta-ethnography [ti]) OR Systematic literature search [ti] OR Systematic literature research [ti] OR meta-analyses [ti] OR metaanalyses [ti] OR metaanalysis [ti] OR meta-analysis [ti] OR meta-analytic review [ti] OR meta-analytical review [ti] OR meta-analysis [pt] OR ((search* [tiab] OR medline [tiab] OR pubmed [tiab] OR embase [tiab] OR Cochrane [tiab] OR scopus [tiab] or web of science [tiab] OR sources of information [tiab] OR data sources [tiab] OR following databases [tiab]) AND (study selection [tiab] OR selection criteria [tiab] OR eligibility criteria [tiab] OR inclusion criteria [tiab] OR exclusion criteria [tiab])) OR systematic review [pt]) | 362,214 |
| 3 | "HIV infections"[Mesh] OR AIDS[Mesh] OR "HIV Seropositivity"[Mesh] OR "AIDS Serodiagnosis"[Mesh] OR "HIV infect*"[tiab] OR "HIV positiv*"[tiab] OR "HIV care"[tiab] OR (HIV[tiab] AND incidence[tiab]) OR (HIV[tiab] AND prevent*[tiab]) OR (HIV[tiab] AND risk*[tiab]) OR (HIV[tiab] AND prevalen*[tiab]) OR (HIV[tiab] AND "new* infect*"[tiab]) OR (HIV[tiab] AND "new* diagnos*"[tiab]) OR (HIV[tiab] AND transm*[tiab]) OR (living[tiab] AND HIV[tiab]) OR (living[tiab] AND (AIDS[tiab] NOT hearing[tiab])) | 362,184 |
| 4 | #1 AND #2 AND #3 | 492 |
| 5 | #4 NOT (letter [pt] OR editorial [pt] OR comment [pt] OR case reports [pt] OR historical article [pt] OR report [ti] OR protocol [ti] OR protocols [ti] OR withdrawn [ti] OR retraction of publication [pt] OR retraction of publication as topic [mesh] OR retracted publication [pt] OR reply [ti] OR published erratum [pt]) | **473** |

**CINAHL**

| Search | Query | # of Results |
| --- | --- | --- |
| 1 | (TI MSM OR AB MSM) OR (TI gay OR AB gay) OR (TI homosexual* OR AB homosexual*) OR (MH "Gay Persons") OR (MH "Gay Men") OR (MH "LGBTQ+ Persons+") OR (MH "Homosexuality") OR (MH "Men Who Have Sex With Men") OR (MH "Bisexuality") OR (TI "men who have sex with men" OR AB "men who have sex with men") OR (TI bisexual* OR AB bisexual*) | 29,851 |
| 2 | ((TI systematic* AND TI review) OR TI "Systematic overview*" OR TI "Cochrane review*" OR TI "systemic review*" OR TI "scoping review" OR TI "scoping literature review" OR TI "mapping review" OR TI "Umbrella review*" OR (TI "review of reviews" OR TI "overview of reviews") OR TI meta-review OR (TI "integrative review" OR TI "integrated review" OR TI "integrative overview" OR TI meta-synthesis OR TI metasynthesis OR TI "quantitative review" OR TI "quantitative synthesis" OR TI "research synthesis" OR TI meta-ethnography) OR TI "Systematic literature search" OR TI "Systematic literature research" OR TI meta-analyses OR TI metaanalyses OR TI metaanalysis OR TI meta-analysis OR TI "meta-analytic review" OR TI "meta-analytical review" OR (((TI search* OR AB search*) OR (TI medline OR AB medline) OR (TI pubmed OR AB pubmed) OR (TI embase OR AB embase) OR (TI Cochrane OR AB Cochrane) OR (TI scopus OR AB scopus) OR (TI "web of science" OR AB "web of science") OR (TI "sources of information" OR AB "sources of information") OR (TI "data sources" OR AB "data sources") OR (TI "following databases" OR AB "following databases")) AND ((TI "study selection" OR AB "study selection") OR (TI "selection criteria" OR AB "selection criteria") OR (TI "eligibility criteria" OR AB "eligibility criteria") OR (TI "inclusion criteria" OR AB "inclusion criteria") OR (TI "exclusion criteria" OR AB "exclusion criteria")))) | 148,947 |
| 3 | (MH "Human Immunodeficiency Virus+") OR (MH "HIV Seropositivity") OR (MH "HIV-Positive Persons+") OR (MH "HIV Infections+") OR (MH "AIDS Serodiagnosis") OR (MH "AIDS Patients") OR (MH "AIDS-Related Complex") OR (MH "HIV-AIDS Nursing") OR (MH "Acquired Immunodeficiency Syndrome") OR TI (HIV infect*) OR TI (HIV positiv*) OR TI (HIV care) OR TI (HIV N4 incidence) OR TI (HIV N4 prevent*) OR TI (HIV N4 risk*) OR TI (HIV N4 prevalen*) OR TI (HIV N4 new* infect*) OR TI (HIV N4 new* diagnos*) OR TI (HIV N4 transm*) OR TI (living N4 HIV) OR TI (living N4 (AIDS NOT hearing) ) OR AB (HIV infect*) OR AB (HIV positiv*) OR AB (HIV care) OR AB (HIV N4 incidence) OR AB (HIV N4 prevent*) OR AB (HIV N4 risk*) OR AB (HIV N4 prevalen*) OR AB (HIV N4 new* infect*) OR AB (HIV N4 new* diagnos*) OR AB (HIV N4 transm*) OR AB (living N4 HIV) OR AB (living N4 (AIDS NOT hearing) ) | 121,634 |
| 4 | S1 AND S2 AND S3 | **226** |

**Web of Science**

| Search | Query | # of results |
| --- | --- | --- |
| 1 | (TI=MSM OR AB=MSM) OR (TI=gay OR AB=gay) OR (TI=homosexual* OR AB=homosexual*) OR ALL=homosexuality OR ALL="homosexuality, male" OR (TI="men who have sex with men" OR AB="men who have sex with men") OR (TI=bisexual* OR AB=bisexual*) OR ALL=bisexuality | 63,907 |
| 2 | (TI="integrative review" OR TI="integrated review" OR TI="integrative overview" OR TI=meta-synthesis OR TI=metasynthesis OR TI="quantitative review" OR TI="quantitative synthesis" OR TI="research synthesis" OR TI=meta-ethnography) OR TI="Systematic literature search" OR TI="Systematic literature research" OR TI=meta-analyses OR TI=metaanalyses OR TI=metaanalysis OR TI=meta-analysis OR TI="meta-analytic review" OR TI="meta-analytical review" OR ALL=meta-analysis OR (((TI=search* OR AB=search*) OR (TI=medline OR AB=medline) OR (TI=pubmed OR AB=pubmed) OR (TI=embase OR AB=embase) OR (TI=Cochrane OR AB=Cochrane) OR (TI=scopus OR AB=scopus) OR (TI="web of science" OR AB="web of science") OR (TI="sources of information" OR AB="sources of information") OR (TI="data sources" OR AB="data sources") OR (TI="following databases" OR AB="following databases")) AND ((TI="study selection" OR AB="study selection") OR (TI="selection criteria" OR AB="selection criteria") OR (TI="eligibility criteria" OR AB="eligibility criteria") OR (TI="inclusion criteria" OR AB="inclusion criteria") OR (TI="exclusion criteria" OR AB="exclusion criteria"))) OR (ALL="systematic review") | 431,766 |
| 3 | ALL="HIV infections" OR ALL=AIDS OR ALL="HIV Seropositivity" OR ALL="AIDS Serodiagnosis" OR (TI="HIV infect?" OR AB="HIV infect?") OR (TI="HIV positiv?" OR AB="HIV positiv?") OR (TI="HIV care" OR AB="HIV care") OR (TI=(HIV NEAR/4 incidence) OR AB=(HIV NEAR/4 incidence)) OR (TI=(HIV NEAR/4 prevent?) OR AB=(HIV NEAR/4 prevent?)) OR (TI=(HIV NEAR/4 risk?) OR AB=(HIV NEAR/4 risk?)) OR (TI=(HIV NEAR/4 prevalen?) OR AB=(HIV NEAR/4 prevalen?)) OR (TI=(HIV NEAR/4 "new? infect?") OR AB=(HIV NEAR/4 "new? infect?")) OR (TI=(HIV NEAR/4 "new? diagnos?") OR AB=(HIV NEAR/4 "new? diagnos?")) OR (TI=(HIV NEAR/4 transm?) OR AB=(HIV NEAR/4 transm?)) OR (TI=(living NEAR/4 HIV) OR AB=(living NEAR/4 HIV)) OR (TI=(living AND (AIDS NOT hearing)) OR AB=(living AND (AIDS NOT hearing))) | 672,006 |
| 4 | #1 AND #2 AND #3 | 345 |
| 5 | #4 NOT (ALL=letter OR ALL=editorial OR ALL=comment OR ALL="case reports" OR ALL="historical article" OR TI=report OR TI=protocol OR TI=protocols OR TI=withdrawn OR ALL="retraction of publication" OR ALL="retraction of publication as topic" OR ALL="retracted publication" OR TI=reply OR ALL="published erratum") | **330** |

**Global Health**

| Search | Query | # of Results |
| --- | --- | --- |
| 1 | (MSM or gay or homosexual*).tw. or exp homosexual men/ or homosexuality/ or bisexual*.tw. or exp bisexual men/ or bisexual men.tw. or bisexuality.sh. or exp men who have sex with men/ or "men who have sex with men".tw. | 20,885 |
| 2 | ((systematic* and review) or "Systematic overview*" or "Cochrane review*" or "systemic review*" or "scoping review" or "scoping literature review" or "mapping review" or "Umbrella review*" or ("review of reviews" or "overview of reviews") or meta-review or ("integrative review" or "integrated review" or "integrative overview" or meta-synthesis or metasynthesis or "quantitative review" or "quantitative synthesis" or "research synthesis" or meta-ethnography) or "Systematic literature search" or "Systematic literature research" or meta-analyses or metaanalyses or metaanalysis or meta-analysis or "meta-analytic review" or "meta-analytical review").ti. or ((search* or medline or pubmed or embase or Cochrane or scopus or "web of science" or "sources of information" or "data sources" or "following databases") and ("study selection" or "selection criteria" or "eligibility criteria" or "inclusion criteria" or "exclusion criteria")).tw. | 59,912 |
| 3 | exp HIV infections/ OR exp human immunodeficiency viruses/ OR exp seroprevalence/ OR exp acquired immune deficiency syndrome/ OR (HIV infect$).ti,ab OR (HIV positiv$).ti,ab OR (HIV care).ti,ab OR (HIV adj4 incidence).ti,ab OR (HIV adj4 prevent$).ti,ab OR (HIV adj4 risk$).ti,ab OR (HIV adj4 prevalen$).ti,ab OR (HIV adj4 new$ infect$).ti,ab OR (HIV adj4 new$ diagnos$).ti,ab OR (HIV adj4 transm$).ti,ab OR (living adj4 HIV).ti,ab OR (living adj4 (AIDS not hearing)).ti,ab | 220,021 |
| 4 | 1 and 2 and 3 | **267** |

**Scopus**

| Search | Query | # of Results |
| --- | --- | --- |
| 1 | TITLE-ABS-KEY ( msm ) OR TITLE-ABS-KEY ( gay ) OR TITLE-ABS-KEY ( homosexual* ) OR TITLE-ABS-KEY ( male AND homosexuality ) OR TITLE-ABS-KEY ( "men who have sex with men" ) OR TITLE-ABS-KEY ( bisexual* ) | 95,524 |
| 2 | ( ( TITLE ( systematic* ) AND TITLE ( review ) ) OR TITLE ( "Systematic overview*" ) OR TITLE ( "Cochrane review*" ) OR TITLE ( "systemic review*" ) OR TITLE ( "scoping review" ) OR TITLE ( "scoping literature review" ) OR TITLE ( "mapping review" ) OR TITLE ( "Umbrella review*" ) OR ( TITLE ( "review of reviews" ) OR TITLE ( "overview of reviews" ) ) OR TITLE ( meta-review ) OR ( TITLE ( "integrative review" ) OR TITLE ( "integrated review" ) OR TITLE ( "integrative overview" ) OR TITLE ( meta-synthesis ) OR TITLE ( metasynthesis ) OR TITLE ( "quantitative review" ) OR TITLE ( "quantitative synthesis" ) OR TITLE ( "research synthesis" ) OR TITLE ( meta-ethnography ) ) OR TITLE ( "Systematic literature search" ) OR TITLE ( "Systematic literature research" ) OR TITLE ( meta-analyses ) OR TITLE ( metaanalyses ) OR TITLE ( metaanalysis ) OR TITLE ( meta-analysis ) OR TITLE ( "meta-analytic review" ) OR TITLE ( "meta-analytical review" ) OR TITLE-ABS-KEY ( meta-analysis ) OR TITLE-ABS-KEY ( systematic AND review ) OR ( ( TITLE-ABS ( search* ) OR TITLE-ABS ( medline ) OR TITLE-ABS ( pubmed ) OR TITLE-ABS ( embase ) OR TITLE-ABS ( cochrane ) OR TITLE-ABS ( scopus ) OR TITLE-ABS ( "web of science" ) OR TITLE-ABS ( "sources of information" ) OR TITLE-ABS ( "data sources" ) OR TITLE-ABS ( "following databases" ) ) AND ( TITLE-ABS ( "study selection" ) OR TITLE-ABS ( "selection criteria" ) OR TITLE-ABS ( "eligibility criteria" ) OR TITLE-ABS ( "inclusion criteria" ) OR TITLE-ABS ( "exclusion criteria" ) ) ) OR DOCTYPE ( review ) ) | 663,890 |
| 3 | ( DOCTYPE ( bk ) OR DOCTYPE ( ch ) OR DOCTYPE ( bz ) OR DOCTYPE ( cp ) OR DOCTYPE ( dp ) OR TITLE ( ed ) OR TITLE ( er ) OR TITLE ( le ) OR TITLE ( mm ) OR DOCTYPE ( no ) OR KEY ( "retraction of publication as topic" ) OR DOCTYPE ( tb ) OR TITLE ( reply ) OR DOCTYPE ( pr ) ) | 16,150,765 |
| 4 | TITLE-ABS-KEY ( "HIV infections" ) OR TITLE-ABS-KEY ( aids ) OR TITLE-ABS-KEY ( "HIV Seropositivity" ) OR TITLE-ABS-KEY ( "AIDS Serodiagnosis" ) OR TITLE-ABS-KEY ( "HIV infect?" ) OR TITLE-ABS-KEY ( "HIV positiv?" ) OR TITLE-ABS-KEY ( "HIV care" ) OR TITLE-ABS-KEY ( hiv W/4 incidence ) OR TITLE-ABS-KEY ( hiv W/4 prevent? ) OR TITLE-ABS-KEY ( hiv W/4 risk? ) OR TITLE-ABS-KEY ( hiv W/4 prevalen? ) OR TITLE-ABS-KEY ( hiv W/4 "new? infect?" ) OR TITLE-ABS-KEY ( hiv W/4 "new? diagnos?" ) OR TITLE-ABS-KEY ( hiv W/4 transm? ) OR TITLE-ABS-KEY ( living W/4 hiv ) OR TITLE-ABS-KEY ( living W/4 aids ) | 494,124 |
| 5 | #1 AND #2 AND #4 | 687 |
| 6 | #5 AND NOT #3 | 678 |

**PsycInfo**

| Search | Search String | # of results |
| --- | --- | --- |
| 1 | (TI MSM OR AB MSM) OR (TI gay OR AB gay) OR (TI homosexual* OR AB homosexual*) OR DE "Homosexuality" OR DE "Male Homosexuality" OR DE "Same Sex Intercourse" OR DE "LGBTQ" OR DE "Bisexuality" OR (TI "men who have sex with men" OR AB "men who have sex with men") OR (TI bisexual* OR AB bisexual*) | 44,971 |
| 2 | DE "Retrovirus Infections" OR DE "AIDS" OR DE "HIV" OR TI (HIV infect*) OR TI (HIV positiv*) OR TI (HIV care) OR TI (HIV N4 incidence) OR TI (HIV N4 prevent*) OR TI (HIV N4 risk*) OR TI (HIV N4 prevalen*) OR TI (HIV N4 new* infect*) OR TI (HIV N4 new* diagnos*) OR TI (HIV N4 transm*) OR TI (living N4 HIV) OR TI (living N4 (AIDS not hearing) ) OR AB (HIV infect*) OR AB (HIV positiv*) OR AB (HIV care) OR AB (HIV N4 incidence) OR AB (HIV N4 prevent*) OR AB (HIV N4 risk*) OR AB (HIV N4 prevalen*) OR AB (HIV N4 new* infect*) OR AB (HIV N4 new* diagnos*) OR AB (HIV N4 transm*) OR AB (living N4 HIV) OR AB (living N4 (AIDS not hearing) ) | 55,637 |
| 3 | ((TI systematic* AND TI review) OR TI "Systematic overview*" OR TI "Cochrane review*" OR TI "systemic review*" OR TI "scoping review" OR TI "scoping literature review" OR TI "mapping review" OR TI "Umbrella review*" OR (TI "review of reviews" OR TI "overview of reviews") OR TI meta-review OR (TI "integrative review" OR TI "integrated review" OR TI "integrative overview" OR TI meta-synthesis OR TI metasynthesis OR TI "quantitative review" OR TI "quantitative synthesis" OR TI "research synthesis" OR TI meta-ethnography) OR TI "Systematic literature search" OR TI "Systematic literature research" OR TI meta-analyses OR TI metaanalyses OR TI metaanalysis OR TI meta-analysis OR TI "meta-analytic review" OR TI "meta-analytical review" OR (((TI search* OR AB search*) OR (TI medline OR AB medline) OR (TI pubmed OR AB pubmed) OR (TI embase OR AB embase) OR (TI Cochrane OR AB Cochrane) OR (TI scopus OR AB scopus) OR (TI "web of science" OR AB "web of science") OR (TI "sources of information" OR AB "sources of information") OR (TI "data sources" OR AB "data sources") OR (TI "following databases" OR AB "following databases")) AND ((TI "study selection" OR AB "study selection") OR (TI "selection criteria" OR AB "selection criteria") OR (TI "eligibility criteria" OR AB "eligibility criteria") OR (TI "inclusion criteria" OR AB "inclusion criteria") OR (TI "exclusion criteria" OR AB "exclusion criteria")))) | 55,226 |
| 4 | S1 AND S2 AND S3 | **128** |
