# Supplementary material for: Spiraling Risk: Visualizing the multilevel factors that socially pattern HIV risk among gay, bisexual & other men who have sex with men using Complex Systems Theory
Source: Curr HIV/AIDS Rep. 2023 Jul 24;20(4):206–17. doi: 10.1007/s11904-023-00664-y (PMC10403445; doi:10.1007/s11904-023-00664-y)
Supplement: Supplementary file 2 — Supplementary file2 (DOCX 73 KB) [file 11904_2023_664_MOESM2_ESM.docx]

Table 2. Matrix of study characteristics and risk of bias assessments (n=63*)*

| Title | Author and Year | Population & sample size | Study Designs & # of studies | Geography | Strengths | Risk of Bias | Quality Assessment |
| --- | --- | --- | --- | --- | --- | --- | --- |
| Is pre-exposure prophylaxis effective for preventing HIV infection in men who have sex with men? | Allende 2017 | MSM | RCTs, longitudinal  23 studies included |  | - Searched MEDLINE, EMBASE, and Cochrane databases - Utilized Friendly Summaries of Body of Evidence using Epistemonikos | No risk of bias assessment  # reviewers not specified  Lacking detail on analytical methods  No detail on framework for review | Critically low |
| Does per-act HIV-1 transmission risk through anal sex vary by gender? An updated systematic review and meta-analysis | Baggaley 2018 | MSM  Heterosexual | Longitudinal 4 studies included | United States  Europe  Australia | - Searched Medline, Embase, CINAHL, Web of Science, Global Health, and Cochrane Library - Utilized PRISMA criteria - Performed random effects inverse-variance meta analysis - Arcsin-transformed study estimates that were back transformed - I2 statistic used to assess heterogeneity - Included only randomized controlled trials and longitudinal studies - Two screener, reviewer, extractor and quality assessment system - Detailed reporting on studies |  | High |
| HIV transmission risk through anal intercourse: Systematic review, meta-analysis and implications for HIV prevention | Baggaley 2010 | MSM  Heterosexuals | Longitudinal 27 studies included | Global | - Searched PubMed, Science Direct and NLM Gateway databases - MOOSE guidelines utilized for observational studies - Performed random effects inverse-variance meta-analysis - Studies with sample sizes less than 10 omitted - Detailed reporting on studies | No risk of bias assessment | Moderate |
| Enhancing benefits or increasing harms: Community responses for HIV among men who have sex with men, transgender women, female sex workers, and people who inject drugs | Baral 2014 | MSM  FSW  Transgender women  PWID | Cross-sectional 22 studies included |  | - Two screener, reviewer, extractor and quality assessment system - MeSH terms and search logic provided | Only English language  Only searched in PubMed  No reporting on guidelines or frameworks used  No risk of bias assessment | Critically low |
| Elevated risk for HIV infection among men who have sex with men in low- and middle-income countries 2000-2006: A systematic review | Baral 2007 | MSM | Cross-section 83 studies included al | Low and middle-income countries  83 global articles  38 countries represented  12 European studies included | - Four databases searched, PubMed, EMBASE, EBSCO, and the Cochrane Database of Systematic Reviews - Validity improved as studies with less than n=50 were excluded - Two screener, reviewer, extractor and quality assessment system - Included a meta-analysis with odds ratios and 95% confidence intervals using Mantel-Haenszel method and random-effects models - Conducted heterogeneity testing using DerSimonian and Laird Q test to address type I error - Sensitivity analyses included to remove the estimated GBMSM population from the general population estimate of men - Sensitivity analyses conducted to explore epidemic level and IDU predominance’s role among MSM and HIV - Large aggregate sample of GBMSM (n=63,538) | Majority of studies included were cross-sectional  Two-person review not done in parallel—one was a confirmation reviewer  MeSH terms and search logic provided at basic level | Moderate |
| The effectiveness of MI4MSM: How useful is motivational interviewing as an HIV risk prevention program for men who have sex with men? A systematic review | Berg 2011 | MSM | RCT 13 studies included | United States  The Netherlands | - Searched CENTRAL, CDSR, DARE, EMBASE, ISI Web of Knowledge, MEDLINE, POPLINE, PsycInfo, Sociological Abstracts, Google Scholar, Motivational Interviewing Newsletter - Utilized (S)PICO model for search and screening - Two screener, reviewer, extractor and quality assessment system - Cochrane two-part Risk of Bias tool used - Application of Grading of Recommendations, Assessment Development and Evaluation (GRADE) - Mantel-Haenszel random effects meta-analyses for dichotomous outcomes and inverse-variance random effects meta-analyses for continuous outcomes were used - Heterogeneity was assessed with X2 and I2 tests - RCT inclusion, as compared to no intervention, wait list control, placebo | MeSH search terms and logic available by request to lead author | High |
| Evidence and knowledge gaps on the disease burden in sexual and gender minorities: A review of systematic reviews | Blondeel 2016 | MSM  WSW  Transgender persons | Review of reviews; design of underlying studies not specified 30 studies included | Global | - Utilized PRISMA criteria for the search methods - Shared MESH terms - Searched Cochrane Database of Systematic Reviews and Campbell Collaboration Library of Systematic Reviews, PubMed and Google Scholar - Used AMSTAR scale for bias assessments | No MeSH logic shared | High |
| Sexually transmitted infection as a risk factor for homosexual HIV transmission: A systematic review of epidemiological studies | Bonell 2000 | MSM | Cohort-nested case control 16 studies included | USA | - Searched Medline Express and Embase, and abstracts from International Conferences on AIDS - Provided MeSH terms - Studies that address confounded only included | No MeSH logic shared  Very strict inclusion and exclusion criteria  No risk of bias conducted  # of reviewers not stated | Low |
| The Role of Networks in Racial Disparities in HIV Incidence Among Men Who Have Sex with Men in the United States | Bonett 2020 | MSM | Cross-sectional (n=22), prospective cohort (n=3) 25 studies included | USA | - Searched in PubMed, Scopus, PsycINFO, and Sociological Abstracts - MeSH terms and search logic provided - Relevant inclusion and exclusion criteria | Single coder  No risk of bias conducted | Low |
| A systematic review of parental influences on the health and well-being of lesbian, gay, and bisexual youth: Time for a new public health research and practice agenda | Bouris 2010 | LGB youth  Six articles that examined sexual health outcomes | Cross-sectional (n=26), longitudinal (5) 31 studies included | USA | - Searched in PubMed, PsycINFO, PsycArticles, Web of Science, Social Service Abstracts - Reviewed reference lists of articles - Relevant inclusion and exclusion criteria | Only one prospective study  No information on data extraction  No information on # of reviewers  No MeSH terms or logic shared  No risk of bias conducted | Critically low |
| A Systematic Review up to 2018 of HIV and Associated Factors Among Criminal Justice–Involved (CJI) Black Sexual and Gender Minority Populations in the United States (US) | Brewer 2021 | Black criminal justice involved SGM | Quasi-RCT (n=36), cross-sectional (n=6), longitudinal (n=1), case-control (n-1) 47 studies included | USA | - Searched PubMed, PsycINFO, and SocINDEX databases - Relevant inclusion and exclusion criteria - 10-item risk of bias tool adapted by Hoy et al. for prevalence studies | MeSH terms and logic not shared  Only one screener & reviewer | Low |
| Non-occupational postexposure prophylaxis for HIV: A systematic review | Bryant 2009 | Diverse populations N=200 | Cohort study (n=1) 1 study included | Global | - Searched in Cochrane Database of Systematic Reviews, Cochrane Central Register of Controlled Trials, Medline, EMBASE, PubMEd, NHS Economic Evaluations Database, NHS Technology Assessment database, Database of Abstracts of Reviews of Effectiveness, EconLit, National Research Register, Current Controlled Trials, and ClinicalTrials.gov - Relevant inclusion and exclusion criteria - Two screener, reviewer, extractor and quality assessment system - Utilized criteria for quality screening by Spitzer et al., for economic evaluations a checklist adapted by Drummond and Jefferson and Phillips and colleagues | MeSH terms and logic not shared | High |
| Associations between Intimate Partner Violence and Health among Men Who Have Sex with Men: A Systematic Review and Meta-Analysis | Buller 2014 | MSM N=13,797 | Cross-sectional 13 studies included | Global | - Searched in MEDLINE, EMBASE, Global Health, PsycINFO, HMIC Social Policy and Practice, CINAHL, IBBS, Web of Science, Africa Web, IMSEAR, IMEMR, LILACS - Utilized PRISMA and MOOSE guidelines for search - Relevant inclusion and exclusion criteria - Two screener, reviewer, extractor and quality assessment system - Used STROBE criteria for quality assessments with interrater agreement as k=0.86 - Random effects meta analyses used to calculate pooled ratios for associations - Meta-regressions analyses performed for heterogeneity tests - Heterogeneity was assessed with I2 tests | MeSH terms and logic not shared | High |
| The use of social networking applications of smartphone and associated sexual risks in lesbian, gay, bisexual, and transgender populations: a systematic review | Choi 2017 | MSM | Cross-sectional 13 studies included | USA  China  Hong Kong | - Followed PRISMA guidelines - MeSH search terms included - Searched in Google Scholar - Two screener, reviewer, extractor and quality assessment system - Used Strengthening the Reporting of Observational Studies in Epidemiology for quality assessment | MeSH logic not shared  No information on extraction process  No inclusion or exclusion criteria provided | Moderate |
| Preexposure prophylaxis for the prevention of HIV infection: Evidence report and systematic review for the US preventive services task force | Chou 2019 | Diverse N=55,000 | RCT (n=14), observational (n=8), diagnostic (n=7) 29 studies included | Global | - Searched in Embase, Medline, and Cochrane Library - Relevant inclusion and exclusion criteria - Two screener, reviewer and quality assessment system - Used USPSTF guidelines to assess quality of studies - Conducted pooled meta analyses using DerSimonian and Laird Q random effects models - Heterogeneity was assessed with I2 tests - Sensitivity analyses conducted | One coder extracted information with second coder affirming accuracy  MeSH terms and logic not shared | High |
| The Prevalence of HIV Among Men Who Have Sex With Men (MSM) and Young MSM in Latin America and the Caribbean: A Systematic Review | Coelho 2021 | MSM | Cross-sectional 47 studies included | Latin America  Caribbean | - Registered in PROSPERO - Followed PRISMA guidelines - Searched in Medline, EMBASE, Virtual Health LIbrary - No language restrictions - Relevant inclusion and exclusion criteria - Two screener, reviewer and data extraction system - MeSH terms and search logic provided | No quality assessment performed | Moderate |
| The prevalence of HIV among MSM in China: A large-scale systematic analysis | Dong 2019 | MSM | Cross-sectional 355 studies included | China | - Searched in Cochrane Library, PubMed, EMBASE, Chinese National Knowledge Infrastructure, Wanfang Data - Two screener, reviewer and data extraction system - Provided MeSH terms - Relevant inclusion and exclusion criteria - Conducted pooled meta-analyses using DerSimonian and Laird Q random effects models - Heterogeneity was assessed with Cochran’s Q and I2 tests - Subgroup analyses conducted | Limited MeSH logic provided | High |
| Men who have sex with men (MSM) in public sex environments (PSEs): A systematic review of quantitative literature | Frankis 2005 | MSM | Cross-sectional 8 studies included | Global | - Searched in Medline, BIDS, and Web of Science - Relevant inclusion and exclusion criteria | Only a single coder and reviewer system used  No risk of bias assessment included  No guidelines used for search and review  MeSH terms and logic not shared | Critically low |
| Herpes simplex virus 2 infection increases HIV acquisition in men and women: Systematic review and meta-analysis of longitudinal studies | Freeman 2006 | Men and women  19 studies total, 9 in general populations of men and | Longitudinal  19 studies included, 5 among MSM | Global | - Searched in PubMed and Embase, International AIDS Conference and International Society for Sexually Transmitted Diseases Research Conference databases - MeSH terms provided - Two screener, reviewer and data extraction system - Risk of bias assessments conducted - Meta-analyses conducted using random-effects models adjusted for age and sexual behavior - Conducted tests for heterogeneity - Subgroup analyses conducted by population group | MeSH search logic not provided  No guidelines provided for quality assessments | High |
| HIV infection and sexual risk among men who have sex with men and women (MSMW): A systematic review and meta-analysis | Friedman 2014 | MSMW | Cross-sectional 31 studies included | USA | - Utilized PRISMA guidelines - Searched in PubMed and PsycINFO - Two screener, reviewer and data extraction system - Q statistic used to test heterogeneity - Meta analyses conducted using random and fixed effects models for within and across subgroup analyses - Sensitivity analyses performed for outlier effects - MeSH terms and search logic provided - Relevant inclusion and exclusion criteria - Egger’s test used for publication bias | Only two databases searched | High |
| Review of sexualized drug use associated with sexually transmitted and blood-borne infections in gay, bisexual and other men who have sex with men | Guerra 2020 | Gay, bisexual and MSM | Cross-sectional (n=11), case-control (n=4), cohort (n=2) 19 studies included | North America  Europe | - Searched in MEDLINE, Embase, and CINAHL - Relevant inclusion and exclusion criteria - Two screener, reviewer and data extraction system - Used Meta Quality Appraisal Tool for quality checks - Meta analyses conducted using random effects models - Sensitivity analyses conducted for studies that did not adjusted for condomless sex | No MeSH terms or search logic provided  No guidelines provided for conducting the review  No information about heterogeneity checks | Moderate |
| HIV-related behavioral studies of men who have sex with men in China: A systematic review and recommendations for future research | Guo 2011 | MSM | Cross-sectional 33 studies included | China | - Relevant inclusion and exclusion criteria - Searched in MEDLINE, PubMed, PsycINFO, and Sociological Abstract - MeSH search terms provided | MeSH search logic not provided  No guidelines provided for conducting the review  No information provided on number of reviewers and coders  No inclusion of quality assessments nor quality assessment guidelines | Critically low |
| HIV, sexually transmitted infection, and substance use continuum of care interventions among criminal justice-involved black men who have sex with men: A systematic review | Harawa 2018 | Black MSM criminal justice involved | RCT (n=13), Quasi-RCT (n=6), cross sectional (r=8), other (r=31) 58 studies included | USA | - Searched in PubMed, MEDLINE, Cochrane, CINAHL, and PsycINFO databases - MeSH search terms and logic provided - Relevant inclusion and exclusion criteria - Three screener, reviewer and data extraction system - Utilized Downs and Black quality and bias assessment tool (2-person team) | No guidelines provided for conducting the review | High |
| HIV trends and related risk factors among men having sex with men in mainland China: Findings from a systematic literature review | He 2011 | MSM | Cross-sectional 45 studies included | China | - Searched in PubMed and China National Knowledge Infrastructure - Reviewed China CDC and Ministry of Health reports - MeSH search terms provided - Two screener, reviewer and data extraction system - Relevant inclusion and exclusion criteria - Meta-analyses conducted using random effects models - Heterogeneity was assessed with I2 and Q tests - Egger’s test used for publication bias - Sensitivity analyses conducted for outliers and type of sampling method | MeSH search logic not provided  Only two databases searched | High |
| A narrative systematic review of sexualised drug use and sexual health outcomes among LGBT people | Hibbert 2021 | LGBT | Cross-sectional 75 studies included | Global | - Utilized PRISMA guidelines to conduct review - Searched in MEDLINE, PsycINFO, CINAHL, Plus and Web of Science - Registered protocol in PROSPERO - Relevant inclusion and exclusion criteria - PECO framework used to develop search - Two screener and reviewer system used - One person conducted data extraction - Effective Public Health Practice Project Quality Assessment Tool and Center for Evidence-Based Management critical appraisal checklist for quality assessments | Second reviewer checked extraction | High |
| Human papillomavirus infection and increased risk of HIV acquisition. A systematic review and meta-analysis | Houlihan 2012 | MSM  Heterosexual men  Heterosexual women  N=12,750 | Cohort (n=7), nested case control (n=1) 18 studies included | Global, mostly Africa | - Searched in PubMed, EMBASE and international HIV conference abstracts - Two screener and reviewer system used - Relevant inclusion and exclusion criteria - MeSH search terms and logic provided - Quality and bias assessment completed using a proscribed approach but not guidelines - Meta-analyses conducted using random effects models - Sensitivity analyses conducted to exclude unadjusted analyses from meta-analyses - Begg’s test used to examine publication bias - Heterogeneity was assessed with I2 |  | High |
| Efficacy and safety of oral TDF-based pre-exposure prophylaxis for men who have sex with men: A systematic review and meta-analysis | Huang 2018 | MSM | RCT (n=6), OLE (n=8) 14 studies included | Global | - Reviewed registered in PROSPERO - Utilized PRISMA criteria - Searched in PubMed, Web of Science, Google Scholar, and ClinicalTrials.gov - MeSH search terms and logic provided - Relevant inclusion and exclusion criteria - Two screener, reviewer and data extraction system used - Begg’s test used to examine publication bias - Heterogeneity was assessed with I2 - Subgroup analyses conducted to conduct differences among blood-based adherence levels |  | High |
| Experienced Homophobia and HIV Infection Risk among U.S. Gay, Bisexual, and Other Men Who Have Sex with Men: A Meta-Analysis | Jeffries 2021 | Gay, bisexual and MSM | Cross-sectional (n=42), prospective (n=2) 44 studies included | USA | - Utilized PRIMSA criteria to conduct review - Searched in Embase, MEDLINE, PsycINFO, and Sociological Abstracts - MeSH search terms provided - Relevant inclusion and exclusion criteria - Three groups of 2-persons data extraction system used that each reviewed 1/3 of articles - Meta-analyses conducted using random effects and inverse variance models - Fixed effect models were used to combine effect sizes across subgroups within studies - Heterogeneity was assessed with I2 - Begg and Mazumdar’s tests used to assess publication bias | MeSH search logic not provided  One screener and reviewer of abstracts and titles  Bivariate analyses used to make comparison easier but loses “causal” claims | Moderate |
| A meta-analysis of the efficacy of HAART on HIV transmission and its impact on sexual risk behaviours among men who have sex with men | Jiang 2020 | MSM N=26,040 | Cross-sectional (n=7), cohort (n=8) 18 studies included | Global, mostly UK, Australia, USA | - Searched in PubMed, ScienceDirect and Google Scholar - MeSH search terms and logic provided - Relevant inclusion and exclusion criteria - Utilized PRISMA criteria to conduct review - Process for data extraction provided - Cohort data used for meta-analyses - Heterogeneity was assessed with Q and I2 tests - Meta-analyses conducted using random effects - Egger’s intercept used to assess publication bias | One person screener, reviewer and data extraction system | Moderate |
| HIV incidence in Asia: A review of available data and assessment of the epidemic | KainneDokubo 2013 | Priority populations | N/A 111 studies included | Asia |  | Methods section entirely missing | Critically low |
| Serosorting and HIV/STI Infection among HIV-Negative MSM and Transgender People: A Systematic Review and Meta-Analysis to Inform WHO Guidelines. | Kennedy 2013 | MSM  Transgender folks | Longitudinal 4 studies included | USA  Australia | - Searched in PubMed, PsycINFO, Sociological Abstracts, CINAHL, and EMBASE - Relevant inclusion and exclusion criteria - MeSH search terms provided - Two person reviewer, screener and data extraction system used - Used GRADE systems for quality assessments - Meta-analyses conducted using random effects models - Heterogeneity was assessed with 2 tests | MeSH search logic not provided  No test for publication bias assessment | High |
| HIV risk behaviours among immigrant and ethnic minority gay and bisexual men in North America and Europe: A systematic review | Lewis 2017 | Immigrant and ethnic minority gay and bisexual men | Cross-sectional 33 studies included | North America  Europe | - Utilized PRISMA guidelines to conduct review - Searched in PubMed - MeSH search terms provided | MeSH search logic not provided  No quality or bias assessment provided  No information on number of persons who screened, reviewed and extracted  Only one database searched | Critically low |
| HIV incidence among men who have sex with men in China: A meta-analysis of published studies | Li 2011 | MSM | Cohort (n=3), cross-sectional (n=9) 12 studies included | China | - Searched in PubMed, China National Knowledge Information, and Chinese Wanfang databases - MeSH search terms provided - Relevant inclusio and exclusion criteria - Two person screener, reviewer and data extraction system used - Quality and bias assessments conducted using an appropriate set of items provided in article - Incidence estimates were calculated according to relevant methods to the study design (e.g., cohort, cross sectional) | MeSH search logic not provided | High |
| Association between rectal douching and HIV and other sexually transmitted infections among men who have sex with men: A systematic review and meta-analysis | Li 2019 | MSM | Cross-sectional (n=23), cohort (n=5) 24 studies included | Global | - Searched in PubMed, EMBASE, Scopus, and Web of Science - Utilized PRISMA criteria to conduct search - Two person screener, reviewer and data extraction system used - Relevant inclusion and exclusion criteria - MeSH search terms provided - Assessed quality using Newcastle-Ottawa Scale - Heterogeneity was assessed with Q and I2 tests - Meta-analyses conducted using random effects - Egger’s intercept used to assess publication bias | MeSH search logic not provided | High |
| HIV risk among men who have sex with men who have experienced childhood sexual abuse: Systematic review and meta-analysis | Lloyd 2012 | MSM N=15,622 | Cross-sectional (n=10), RCT (n=1), longitudinal (n=1) 12 studies included | USA | - Searched in PubMed, MEDLINE, PsycINFO, ERIC, SocIndex, AMED and CINAHL - MeSH search terms provided - Relevant inclusion and exclusion criteria - Two person screener and reviewer system used - Odds ratios computed using Mantel-Haenszel using inverse-variance approach - Chi-square used to measure heterogeneity - STROBE checklist used for quality assessment | MeSH search logic not provided  One person extracted information with the other verifying | Moderate |
| Risk of HIV Acquisition among Men Who Have Sex with Men Infected with Bacterial Sexually Transmitted Infections: A Systematic Review and Meta-Analysis | Malekinejad 2021 | MSM | Prospective cohort (n=18), retrospective cohort (n=6), case control (n=2) 26 studies included | Global | - Registered protocol in PROSPERO - Searched in PubMed, Web of Science, and EMBASE - MeSH search terms and logic provided - Utilized Population, Exposure, Comparator, Outcomes (PECO) schema for study screening & extraction - Used GRADE methods for quality assessment - Utilized PRISMA guidelines to conduct review - Relevant inclusion and exclusion criteria - Two person screener, reviewer and data extraction system used - Meta analyses conducted using random effects models - Heterogeneity was assessed with I2 tests - Sensitivity analyses conducted by setting and methodological characteristics |  | High |
| HIV prevalence among female sex workers, drug users and men who have sex with men in Brazil: A Systematic Review and Meta-analysis | Malta 2010 | MSM  Female sex workers  Drug users | Cross sectional, prospective cohort 8 studies included | Brazil | - Used CONSORT, QUOROM, MOOSE and TREND guidelines for planning and conducting systematic review - Searched in MEDLINE, EMBASE, Cochrane Central, AIDSLINE, AMED, CINAHL, TOXNET, SciELO, Web of Science, intertional HIV and STI conference abstracts, and NIH CRISP - MeSH search terms provided - Two person screener, reviewer and data extraction system used - Meta analyses conducted using DerSimonian-Laird random effects models - Heterogeneity was assessed with I2 tests - Egger’s intercept used to assess publication bias | MeSH search logic not provided | High |
| Relative Risk for HIV Infection Among Men Who Have Sex with Men Engaging in Different Roles in Anal Sex: A Systematic Review and Meta-analysis on Global Data | Meng 2015 | MSM | Cross-sectional, prospective cohort 21 studies included | Global | - Searched in PubMed and Scopus - MeSH search terms provided - Two person screener, reviewer and data extraction system used - Relevant inclusion and exclusion criteria - Utilized PRISMA to conduct review - Meta-analyses conducted by random effects models - Begg’s test used to measure publication bias - Heterogeneity was assessed with I2 tests - Subgroup analyses conducted | MeSH search logic not provided | High |
| Circumcision status and risk of HIV and sexually transmitted infections among men who have sex with men: A meta-analysis | Millett 2008 | MSM | Cross-sectional, cohort 15 studies included | Global | - Searched in MEDLINE, ERIC, Sociofile, PsycINFO, EMBASE, Web of Science, Google Scholar, and international HIV and STI conference abstract databases - Relevant inclusion and exclusion criteria - MeSH search terms provided - Two person screener and reviewer system used - Newcastle-Ottawa Scale used for quality assessments - Meta-analyses conducted with fixed and random effects models using inverse-variance - Heterogeneity was assessed with I2 tests - Egger’s intercept used to assess publication bias - Sensitivity analyses conducted | MeSH search logic not provided  One data extractor and the other reviewed system used | High |
| Comparisons of disparities and risks of HIV infection in black and other men who have sex with men in Canada, UK, and USA: A meta-analysis | Millett 2012 | MSM 106,148 Black MSM 581,777 non-Black MSM | N/A N=194 studies included | Canada  United Kingdom  United States | - Searched in Medline, EMBASE, Google Scholar, and conference abstract databases - MeSH search terms provided - Relevant inclusion and exclusion criteria - Two person data extraction system used - Meta-analyses conducted with random effects models using an inverse-variance weighting | MeSH search logic not provided  No review guidelines provided  No quality/bias assessment provided  No information provided about review and screening processes  No heterogeneity assessment | Low |
| Antiretroviral pre-exposure prophylaxis (PrEP) for preventing HIV in high-risk individuals. | Okwundu 2012 | Priority populations, MSM accounted for 21% of whole sample | RCT 12 studies included | Global | - Relevant inclusion and exclusion criteria - Searched in MEDLINE, Cochrane Central, EMBASE, WHO Clinical Trials, and ClinicalTrials.gov - MeSH search terms and logic provided - Two person screener, reviewer, and data extraction system used - Cochrane Handbook of Systematic Reviews of Interventions guided review - Utilized Cochrane Handbook of Systematic Reviews of Interventions for quality assessment - Meta-analyses performed to provide Mantel-Haenzel odds ratio - Heterogeneity was assessed with I2 tests |  | High |
| Human rights protections and HIV prevalence among MSM who sell sex: Cross-country comparisons from a systematic review and meta-analysis | Oldenburg 2018 | MSM N=31,924 | Cross-sectional 66 studies included | Global | - Searched in PubMed, EMBASE, PsycINFO, Sociological Abstracts, POPLine, CINAHL, and Web of Science, national surveillance systems, and international conference abstract databases - MeSH search terms and logic provided - Relevant inclusion and exclusion criteria - Meta-analyses were conducted using random effects models - Heterogeneity was assessed with I2 and t 2 statistics | No information provided on screening, review, and extraction process  No guidelines used to conduct review  No quality assessment conducted | Moderate |
| Transactional Sex and the HIV Epidemic Among Men Who have Sex with Men (MSM): Results From a Systematic Review and Meta-analysis | Oldenburg 2015 | MSM N=78,121 | Cross-sectional 33 studies included | Global | - Utilized PRISMA to conduct review - Searched in PubMed, EMBASE, PsycINFO, Sociological Abstracts, POPLine, CINAHL, Web of Science, and international conference abstracts - Relevant inclusion and exclusion criteria - MeSH search terms provided - Two person screener, reviewer, and data extraction system used - Meta-analyses were conducted using DerSimonian–Laird random effects models - Heterogeneity was assessed with I2 tests - Egger’s and Begg’s test used to assess publication bias - Used GRADE for quality assessment | MeSH search logic not provided | High |
| Quantifying the Harms and Benefits from Serosorting Among HIV-Negative Gay and Bisexual Men: A Systematic Review and Meta-analysis | Purcell 2017 | Gay and bisexual men | Cross-sectional, Cohort 8 studies included | USA  The Netherlands  Australia | - Searched in MEDLINE, EMBASE, CINAHL, PsycINFO, and Sociological Abstracts - Relevant inclusion and exclusion criteria - MeSH search terms provided - Two person screener, reviewer, and data extraction system used - Quality assessment using eight-point scale used by Kennedy et al. (2013). - Meta-analyses were conducted using random effects models - Heterogeneity was assessed with Q statistic - Sensitivity analyses conducted | MeSH search logic not provided  No test of publication bias presented | High |
| Substance Use and HIV Risk among Men Who Have Sex with Men in Africa: A Systematic Review | Sandfort 2017 | MSM | Cross-sectional 68 studies included | Global | - Searched in MEDLINE, PsycINFO, PubMed, and Web of Science - MeSH search terms provided - Relevant inclusion and exclusion criteria - Two person screener, reviewer, and data extraction system used | MeSH search logic not provided  No framework provided for review  No quality assessment provided  No publication bias assessment | Low |
| Men who have sex with men in india: A systematic review of the literature | Setia 2008 | MSM | Cross-sectional 12 studies included | India | - Searched in Medline, Embase, the Cochrane Library and international conferences - One person screener, reviewer, and data extraction system used - MeSH search terms provided - Conducted quality assessment - Meta-analyses were conducted using random effects models - Heterogeneity was assessed with chi-square statistics | MeSH search logic not provided  No framework for conducting review  No quality assessment guidelines provided  One person reviewer system used  No publication bias assessment conducted | Low |
| Male circumcision for the prevention of human immunodeficiency virus (HIV) acquisition: a meta-analysis | Sharma 2018 | Men | Retrospective/cross-sectional (n=34), RCT (n=3), case-control (n=3), prospective cohort (n=9) 49 studies included | Global | - Reviewed registered in PROSPERO - Utilized PRISMA criteria to conduct review - Relevant inclusion and exclusion criteria - Searched in PubMed, Embase, Cochrane Central Register of Controlled Trials, and ClinicalTrials.gov - Risk of bias assessments performed using Quality Assessment Tool for Quantitative Studies by the Effective Public Health Practice Project - Meta-analyses were conducted using random effects models - Heterogeneity was assessed with I2 tests - Publication bias assessed with funnel plots | No information on how many reviewers, screeners, and data extractors | Moderate |
| HIV testing and engagement with the HIV treatment cascade among men who have sex with men in Africa: a systematic review and meta-analysis | Stannah 2019 | MSM N=44,993 | Cross-sectional (n=64), cohort (n=10) 75 studies included | Africa | - Utilized PRISMA criteria and MOOSE guidelines to conduct review - Searched in Embase, MEDLINE, Scopus, Global Health, and Web of Science - Relevant inclusion and exclusion criteria - Three person screener, reviewer, and data extraction system used - Meta-analyses were conducted using random effects models - Heterogeneity was assessed with I2 tests - AXIS tool for appraising cross-sectional studies - Egger’s test used to assess publication bias |  | High |
| A systematic review of evidence to inform HIV prevention interventions among men who have sex with men in Europe | Stromdahl 2015 | MSM | RCT, Cohort 24 studies included | Europe | - Searched in PubMed, EMBASE, Medline, CINAHL, PsycINFO, Cochrane Library and the World Health Organization publication databases - MeSH search terms provided - Population, Intervention, Comparison, Outcome (PICO) model used for search - Grading of Recommendations Assessment, Development, and Evaluation (GRADE) and HASTE quality assessment used. - Hill’s criteria for causality used in the HASTE grading system. - Two person screener, reviewer, and data extraction system used | MeSH search logic not provided | High |
| Amphetamine-type stimulants and HIV infection among men who have sex with men: Implications on HIV research and prevention from a systematic review and meta-analysis | Vu 2015 | MSM | Cross-sectional, case control, longitudinal 35 studies included | Global | - Searched in MEDLINE, EMBASE, Global Health and PsycINFO - Relevant inclusion and exclusion criteria - MeSH search terms and logic provided - Quality assessments conducted using criteria from Boyle et al. For cross-sectional studies and Wells et al., for case control & longitudinal - Two person screener, reviewer, and data extraction system used - Meta-analyses were conducted using DerSimonian–Laird random effects models - Subgroup analyses were performed - Adjusted meta regression conducted - Sensitivity analyses performed - Egger’s test used to assess publication bias | No framework provided for the review | High |
| Sexual Risk Behaviors and HIV Infection among Men Who Have Sex with Men and Women in China: Evidence from a Systematic Review and Meta-Analysis | Wang 2015 | MSM N=19,730 MSMW N=53,536 MSMO | Cross-sectional 36 studies included | China | - PRISMA criteria used for search - Searched in PubMed, Web of Knowledge, Google Scholar, Chinese National Knowledge Infrastructure, VIP, and Wanfang Data - MeSH search terms included - Relevant inclusion and exclusion criteria - Two person screener, reviewer, and data extraction system used - Quality assessment checklist for observational studies used for quality assessment - Meta-analyses performed using random or fixed effects (heterogeneity) - Heterogeneity was assessed with Q and I2 tests - Subgroup analyses were performed - Sensitivity analyses performed - Egger’s test used to assess publication bias | MeSH search logic not provided | High |
| The use of geosocial networking smartphone applications and the risk of sexually transmitted infections among men who have sex with men: a systematic review and meta-analysis. | Wang 2018 | MSM | Cross-sectional  30 studies included | Global | - PRISMA criteria used for search - Searched in Medline - MeSH search terms included - Relevant inclusion and exclusion criteria - Three person screener, reviewer, and data extraction system used - Meta-analyses performed using random or fixed effects model - Heterogeneity was assessed with Q and I2 tests - Begger’s and Egger’s test used to assess publication bias - Sensitivity analyses performed | MeSH search logic not provided  Only one database searched | Moderate |
| HIV Nonoccupational Postexposure Prophylaxis among Men Who Have Sex with Men: A Systematic Review and Meta-Analysis of Global Data | Wang 2020 | MSM | Cross sectional (n=36), cohort (n=14), intervention (n=7)  74 studies included |  | - PRISMA and MOOSE criteria used for search - Searched in PubMed, Scopus, Embase, Cochrane Library, and Web of Science - MeSH search terms provided - Relevant inclusion and exclusion criteria - Two person screener, reviewer, and data extraction system used - Quality assessment conducted using Agency of Healthcare Research and Quality criteria for observational studies and Newcastle-Ottawa scale for cohort studies - Meta-analyses were conducted using DerSimonian–Laird random effects models - Heterogeneity was assessed with I2 tests - Multivariate meta regression conducted - Egger’s test used to assess publication bias - Sensitivity analyses conducted | MeSH search logic not provided | High |
| Uncovering the epidemic of HIV among men who have sex with men in Central Asia | Wirtz 2013 | MSM | Cross-sectional 43 studies included | Central Asia | - Searched in PubMed and EMBASE - MeSH search terms and logic provided | One person coder, reviewer, and data extraction system used with second coder randomly checking 25%  No search criteria provided  No quality assessment  Inclusion and exclusion criteria not explicit | Critically low |
| HIV and Viral Hepatitis among Imprisoned Key Populations | Wirtz 2018 | Persons who are incarcerated | Cross-sectional 43 studies included | Global | - Searched in PubMed, EMBASE, CINAHL, and Criminal Justice Abstracts - MeSH search terms and logic provided - Two person screener, reviewer, and data extraction system used - Relevant inclusion and exclusion criteria - Meta-analyses were conducted using random effects models - Heterogeneity was assessed with I2 tests | No publication bias assessment conducted  No quality assessment provided | Moderate |
| Male circumcision for prevention of homosexual acquisition of HIV in men. | Wiysonge 2011 | Men N=71,69 | Cohort (6), case-control (1), cross-sectional (14) 21 studies included | Global | - Searched in PubMed, EMBASE, the Cochrane Central Register of Controlled Trials, ClinicalTrials.gov, the WHO International Clinical Trials Registry Platform, and international conference abstracts - MeSH search terms and logic provided - Four person screener and reviewer system used - Two person data abstraction system used - Calculated the natural logarithm of the odds ratio and its standard error for each study & then expressed each study result as an odds ratio (OR) with its 95% CI using inverse variance - Heterogeneity was assessed with I2 tests - Quality assessment of publications conducted - Subgroup analyses conducted | No publication bias assessment conducted | High |
| Identifying Resilience Resources for HIV Prevention Among Sexual Minority Men: A Systematic Review | Woodward 2017 | Sexual minority men | N/A 20 studies included | Global | - PRISMA criteria used for search - Searched in Medline, PyscINFO, PsycArticles - Relevant inclusion exclusion criteria - MeSH search terms and logic provided - Six person thematic coding team to identify major themes from review | One person screener, reviewer, and data extraction system used  No quality assessment performed | Moderate |
| Effect of syphilis infection on HIV acquisition: A systematic review and meta-analysis | Wu 2021 | Diverse, majority of studies 16/19 were among MSM N=65,232 | Cohort, Case-Control 22 studies included | Global | - PRISMA criteria used for search - Searched in PubMed, Embase, MEDLINE, Web of Science and Cochrane Library - Relevant inclusion exclusion criteria - MeSH search terms provided - Two person screener, reviewer, and data extraction system used - Utilized Newcastle-Ottawa Scale guidelines for quality assessments - Meta-analyses were conducted using DerSimonian–Laird random effects models - Heterogeneity was assessed with I2 tests - Subgroup analyses conducted - Egger’s test used to assess publication bias | MeSH search logic not provided | High |
| Circumcision to prevent HIV and other sexually transmitted infections in men who have sex with men: a systematic review and meta-analysis of global data | Yuan 2019 | MSM N=119,248 | Cross-sectional (n-45), cohort (n=15), case-control (n=2) 62 studies included | Global | - PRISMA and MOOSE criteria used for search - Searched in PubMed, Web of Science, BioMed Central, Scopus, Research Gate, Cochrane Library, EMBASE, PsycINFO, Google Scholar, and five international HIV/STI conferences - Relevant inclusion and exclusion criteria - MeSH search terms provided - Two person screener, reviewer, and data extraction system used - Utilized Newcastle-Ottawa Scale guidelines for quality assessments and a scale developed by Downs et al. For interventional studies - Meta-analyses were conducted using random effects models - Heterogeneity was assessed with I2 tests - Subgroup analyses performed - Sensitivity analyses performed - Egger’s test used to assess publication bias | MeSH search logic not provided  Unable to perform multivariate meta regression due to study variability | High |
| Voluntary medical male circumcision and HIV infection among men who have sex with men: Implications from a systematic review. | Zhang 2019 | MSM N=117,293 | Cross-sectional (n=24), cohort (n=9) 37 articles included | Global | - PRISMA criteria used for search - MeSH search terms and logic provided - Relevant inclusion and exclusion criteria - Two person screener, reviewer, and data extraction system used - Quality assessments completed of studies - Meta-analyses were conducted using random effects models - Heterogeneity was assessed with I2 tests - Subgroup analyses performed - Sensitivity analyses performed - Funnel plots used to assess publication bias | Databases searched not provided  No framework provided for quality assessments | Moderate |
| HIV incidence and associated risk factors in men who have sex with men in Mainland China: An updated systematic review and meta-analysis | Zhang 2016 | MSM | Prospective cohort 25 studies included | China | - Searched in China National Knowledge Infrastructure (CNKI), Chinese Scientific Journals Full text Database (VIP), Wanfang Database, Google Scholar, Web of Science and PubMed - Relevant inclusion and exclusion criteria - MeSH search terms provided - Strength in that they only included prospective cohort design - Two person screener, reviewer, and data extraction system used - Utilized Newcastle-Ottawa Scale guidelines for quality assessments - Meta-analyses were conducted using random or fixed effects models depending on heterogeneity - Heterogeneity was assessed with Q and I2 tests - Begg’s and Mazumdar rank correlation used to assess publication bias | MeSH search logic not provided  No information provided on guidelines of review | High |
| Association of nitrite inhalants use and unprotected anal intercourse and HIV/syphilis infection among MSM in China: A systematic review and meta-analysis | Zhang 2020 | MSM N=18,981 | Cross-sectional 15 studies included | China | - PRISMA criteria used for search - Registered on PROSPERO - Searched in PubMed, Web of Science, Chinese National Knowledge Infrastructure, Chinese Wanfang Data, and VIP Chinese Journal Database - MeSH search terms and logic provided - Relevant inclusion and exclusion criteria - Two person screener, reviewer, and data extraction system used - Quality assessments performed using quality assessment tool for systematic reviews of observational studies (QATSO) score - Meta-analyses were conducted using random or fixed effects models depending on heterogeneity - Heterogeneity was assessed with I2 tests - Egger’s test used to assess publication bias |  | High |
| Characteristics of Men Who Have Sex With Men Who Use Smartphone Geosocial Networking Applications and Implications for HIV Interventions: A Systematic Review and Meta-Analysis | Zou 2017 | MSM | Cross-sectional 17 studies included | Global | - PRISMA criteria used for search - Searched in Medline and Scopus - MeSH search terms provided - Relevant inclusion and exclusion criteria - Two person screener, reviewer, and data extraction system used - Quality assessment of studies conducted using an eight item checklist - Meta-analyses were conducted using random effects models - Heterogeneity was assessed with I2 and tau 2 tests - Begg’s tested used to assess publication bias | MeSH search logic not provided  No sensitivity tests | High |
