# Supplementary material for: Spiraling Risk: Visualizing the multilevel factors that socially pattern HIV risk among gay, bisexual & other men who have sex with men using Complex Systems Theory
Source: Curr HIV/AIDS Rep. 2023 Jul 24;20(4):206–17. doi: 10.1007/s11904-023-00664-y (PMC10403445; doi:10.1007/s11904-023-00664-y)
Supplement: Supplementary file 3 — Supplementary file3 (DOCX 72 KB) [file 11904_2023_664_MOESM3_ESM.docx]

**Table 3. Matrix of each review’s findings and thematic summaries (n=49)**

| **Title** | **Author** | **Study Designs & # of Studies Included** | **Main findings related to HIV infection or risk** | **Theme** |
| --- | --- | --- | --- | --- |
| Does per-act HIV-1 transmission risk through anal sex vary by gender? An updated systematic review and meta-analysis | Baggaley 2018 | Longitudinal  4 studies | - Pooled estimate of per-act condomless receptive anal sex HIV risk 1.25% 95% CI (0.55-2.23) - Pooled estimate was lower among MSM populations 0.75% 95% CI (0.56-0.98) - Pooled estimate of per-act condomless insertive anal sex HIV risk 0.17% 95% CI (0.09, 0.26)--all studies in MSM - Higher estimated ART use among sexual partners reduced per-act HIV risk | Condomless sex increased HIV risk  Insertive anal sex increased HIV risk  Use of ART, despite condomless sex, reduced HIV risk |
| HIV transmission risk through anal intercourse: Systematic review, meta-analysis and implications for HIV prevention | Baggaley 2010 | Longitudinal  27 studies | - Per-act summary estimate was 1.4% (95% CI 0.2–2.5) or 1.8% (95% CI 0.3–3.2) if Halperin et al.’s abstract estimate is excluded due to lack of further detail on methods. - Per-partner summary estimate 40.4% (95% CI 6.0–74.9) - No significant differences in per-act URAI estimates between heterosexual couples and MSM were found (P ¼ 0.674). - MSM estimates were similar to each other (Q ¼ 0.2; P ¼ 0.635; I 2¼ 0%), heterosexual estimates were heterogeneous (Q ¼ 10.5; P ¼ 0.001; I 2¼ 90%). - Most per-partner estimates were derived from studies on MSM - No evidence that the heterosexual combined URAI–UIAI crude per-partner estimate was significantly different from the eight crude estimates from MSM (P ¼ 0.821) | Unprotected receptive anal sex is associated increased risk of HIV  No difference in risk of receptive anal sex was detected between MSM and heterosexuals |
| Elevated risk for HIV infection among men who have sex with men in low- and middle-income countries 2000-2006: A systematic review | Baral 2007 | Cross-sectional  22 studies | - GBMSM had OR=19.3 (95% CI 18.8-19.8) of HIV than general populations. - Very low prevalence countries had the highest OR of HIV infection among GBMSM [OR=58.4, 95% CI (56.3, 60.6)], as compared to low prevalence [OR=14.4, 95% CI (13.8, 14.9)] and high prevalence countries [OR=9.6, 95% CI (8.9, 10.2)]. - Low-income countries had lower OR for HIV among GBMSM [OR=7.8, 95% CI (7.2, 8.40] than middle income countries [OR=23.4, 95% CI (22.8, 24.0)]. | Risk of HIV was highest in countries with very low HIV prevalence compared to low prevalence and high prevalence countries  HIV risk was lower in low-income countries |
| The effectiveness of MI4MSM: How useful is motivational interviewing as an HIV risk prevention program for men who have sex with men? A systematic review | Berg 2011 | RCT  13 studies | - Metanalysis on HIV acquisition could not be conducted given only one study used HIV as the endpoint - In the one study, estimated odds ratio for HIV infection was 0.84 (95% CI = 0.66, 1.08) or 15.7% (adjusted for baseline covariates) lower in the motivational interviewing group as compared to control group - None of the eight follow-up times (up to 48 months postbaseline) was the difference statistically significant. | Motivational interviewing did not show significant effects on lowering risk of HIV infection |
| Evidence and knowledge gaps on the disease burden in sexual and gender minorities: A review of systematic reviews | Blondeel 2016 | Review of reviews; design of underlying studies not specified  30 studies | - 9/30 reviews provided data on HIV infection; 25/30 on MSM - All reviews showed a high burden of HIV in MSM. - Caribbean highest HIV prevalence in MSM (25.4 %), followed by sub-Saharan Africa with 17.9 % [24]. - Some countries in the Middle East and North Africa show very low HIV prevalence in MSM - MSM in low-middle income countries had a risk of HIV infection that was 19.3 times higher than the general population - Highest HIV prevalence among MSM in the Caribbean | HIV risk was significantly higher in low-middle income countries  There was geographic variation in prevalence of HIV |
| Non-occupational postexposure prophylaxis for HIV: A systematic review | Bryant 2009 | Cohort study  1 study | - Only one published study that met criteria and focused on MSM with low quality that did not include a statistical test for difference between PEP and non-PEP groups | No evidence on the protective effect of PEP |
| Associations between Intimate Partner Violence and Health among Men Who Have Sex with Men: A Systematic Review and Meta-Analysis | Buller 2014 | Cross-sectional  13 studies | - Among studies that included 8,835 MSM, exposure to intimate partner violence was associated with a positive HIV status AOR=1.5, 95% CI (1.3, 1.7). - High consistency between pooled estimates—low heterogeneity I^2^ =0% | Exposure to abuse was associated with risk of HIV infection |
| The use of social networking applications of smartphone and associated sexual risks in lesbian, gay, bisexual, and transgender populations: a systematic review | Choi 2017 | Cross-sectional  13 studies | - Among app users, the prevalence of HIV was elevated as compared to non-app users | Use of apps may increase risk of HIV infection |
| Preexposure prophylaxis for the prevention of HIV infection: Evidence report and systematic review for the US preventive services task force | Chou 2019 | RCT, Observational  29 studies | - PrEP was associated with reduced risk of HIV infection vs placebo or no PrEP (11 trials [n = 18 172]; RR, 0.46 [95% CI, 0.33- 0.66]), but statistical heterogeneity was present (I 2 = 67%) - A stratified analysis found a significant interaction (P < .001) between level of adherence (≤40%, >40 to<70%, ≥70%) and HIV infection - PrEP was effective across HIV risk categories (persons at risk because of heterosexual contact, men who have sex with men, or PWID; P = .43 for interaction) - PrEP was more effective in trials conducted in the United States, Europe, or Canada (3 trials [n = 1323]; RR, 0.13 [95% CI, 0.05- 0.32]; I 2 = 0%)18,31,33 than in trials conducted in Africa, Asia, or internationally (8 trials [n = 16 849]; RR, 0.54 [95% CI, 0.37- 0.79]; I 2 = 72%; P = .004 for interaction) - All trials conducted in the United States, Europe, or Canada reported high adherence and enrolled men who have sex with men. | PrEP use reduced risk of HIV infection  Greater PrEP adherence further reduced risk of HIV infection |
| The Prevalence of HIV Among Men Who Have Sex With Men (MSM) and Young MSM in Latin America and the Caribbean: A Systematic Review | Coelho 2021 | Cross-sectional  47 studies | - Among MSM, older age, sexual risk behaviors, lower socioeconomic status and prior violence experiences all associated with higher prevalence | Riskier sexual behaviors (several partners, sex work, condomless sex, casual sex) increased risk of HIV infection  Lower SES associated with elevated risk of HIV infection  Experience of abuse associated with elevated risk of HIV infection  Older age associated with elevated risk of HIV infection |
| The prevalence of HIV among MSM in China: A large-scale systematic analysis | Dong 2019 | Cross-sectional  355 studies | - HIV prevalence decreases with increasing years of education - MSM seeking sex partners in bathhouses had highest prevalence of HIV (13.4, 95% CI: 10.3-17.1%, N = 22) - Debut with male sexual partner vs. Female sexual partner associated with lower odds of HIV 0.6 (95% CI: 0.5-0.7) - MSM who never used condoms during sex with men, during commercial sex with men, and during sex with a woman in the past 6 months had higher risk of HIV infection, as compared to those who do not use condoms, 0.11 (95% CI: 0.08-0.14), 0.11 (95% CI: 0.09-0.13), and 0.07 (95% CI: 0.03-0.14), respectively - Drug use was not a significant contributor to HIV transmission among Chinese MSM | Higher level of education was associated with lower risk of HIV infection  Public sex associated with higher HIV risk |
| Herpes simplex virus 2 infection increases HIV acquisition in men and women: Systematic review and meta-analysis of longitudinal studies | Freeman 2006 | Longitudinal  19 studies | - Effect of HSV-2 infection on HIV acquisition among MSM was RR=1.7 95% CI (1.2-2.4) | HSV-2 infection associated with higher risk of HIV infection |
| HIV infection and sexual risk among men who have sex with men and women (MSMW): A systematic review and meta-analysis | Friedman 2014 | Cross-sectional  31 studies | - MSM who also have sex with women had lower prevalence, as compared to MSM who only have sex with men, 17% vs. 33% (OR=0.4, 95% CI (0.3, 0.5)) - MSM who also have sex with men had lower HIV prevalence, as compared to men who only had sex with women, 18% vs. 4% (OR=5.7, 95% CI (3.5, 9.4)) - Among men who have sex with men only, higher HIV prevalence was found in studies with greater than 90% minorities (56.4% vs. 26.4%; Q-statistic = 12.8, P,.001) - Among men who have sex with men and women, HIV prevalence was higher in studies with greater than 90% minorities (32.7% vs. 13.2%; Q-statistic = 7.7, P,.01); and by locale: higher HIV prevalence in studies undertaken in the 12 CDC-defined high HIV/AIDS incidence locales (20.9% vs. 10.1%; Q-statistic = 5.4, P,.05) - HIV prevalence among MSMW and MSMO (15.3% vs. 24.0%, respectively) pre-2000 were significantly more convergent than surveys after 2000 (19.4% vs. 46.7%) | MSM who only have sex with men only (compared to MSM who also have sex with women) were at higher risk of HIV infection |
| Review of sexualized drug use associated with sexually transmitted and blood-borne infections in gay, bisexual and other men who have sex with men | Guerra 2020 | Cross-sectional (n=11), case-control (n=4), cohort (n=2)  19 studies | - Three crude OR estimates for the association between SDU and HIV diagnosis, resulting in a pooled crude OR of 3.55 (95%CI 2.19, 5.74) - Two of the above three studies reported adjusted ORs resulting in a pooled adjusted OR of 3.02 (95%CI 0.96, 9.53) - Comparing the crude and adjusted estimates for the two studies that had both estimates for HIV, the ORs were 3.85 (95%CI 1.79, 8.31) and 3.02 (95%CI 0.96, 9.53) | Drug use during sex increased risk of HIV infection |
| HIV, sexually transmitted infection, and substance use continuum of care interventions among criminal justice-involved black men who have sex with men: A systematic review | Harawa 2018 | RCT (n=13), Quasi-RCT (n=6), cross sectional (r=8), other (r=31)  58 studies | - Among men who are incarcerated, studies comparing opt-in to opt-out approaches to HIV testing indicated that opt-out programs result in greater numbers of people screened and new HIV diagnoses uncovered - HIV prevention interventions portrayed the importance of including provision of linkage to social services - Studies suggest that in custody programs might be less effective than those incorporating the reentry to community period - Increasing HIV testing, treatment, and retention in care among GBMSM aged 18 to 54 years in the community and the criminal justice system could result in a 15% and 19% decline in new community- and jail-acquired cases over 10 years | Continuity in services reduced risk of HIV infection among MSM experiencing incarceration |
| HIV trends and related risk factors among men having sex with men in mainland China: Findings from a systematic literature review | He 2011 | Cross-sectional  45 studies | - Two out of three studies found that HIV infection was associated with the number of lifetime male sex partners - Studies indicated that that HIV infection was associated with having anal sex with a male partner in the past 6 months | Number of sexual partners increased risk of HIV infection  Anal sex was associated with increased risk of HIV |
| A narrative systematic review of sexualised drug use and sexual health outcomes among LGBT people | Hibbert 2021 | Cross-sectional  75 studies | - Among the 35 studies that investigated HIV prevalence, 10 used HIV tests and the remaining were self-report - 70% of studies found a bivariate association between poppers use and elevated HIV prevalence - 77% of studies found bivariate associations between chemsex drug use and higher HIV prevalence - Four studies were found that investigated either GHB/GBL or ketamine and two found bivariate associations with HIV prevalence (50%) | Use of nitrites was associated with elevated risk of HIV infection  Use of drugs during sex was associated with elevated risk of HIV infection |
| Human papillomavirus infection and increased risk of HIV acquisition. A systematic review and meta-analysis | Houlihan 2012 | Cohort (n=7), nested case control (n=1)  18 studies | - Only one study examined HPV and HIV infection among MSM - In the one study, multivariable analysis indicated that infection with one HPV type compared with no HPV infection was not significantly associated with HIV acquisition in MSM (aHR=2.0 (95%CI=0.61-6.5)). - Presence of infection with 2 or more HPV types compared with being HPV un-infected was associated with HIV acquisition (aHR=3.5 (95%CI=1.2-10.6)) | HPV infection was associated with increased risk of HIV infection |
| Efficacy and safety of oral TDF-based pre-exposure prophylaxis for men who have sex with men: A systematic review and meta-analysis | Huang 2018 | RCT (n=6), OLE (n=8)  14 studies | - Event rate of HIV among MSM taking PrEP was 1.1% (95% CI 0.6-2.0) - Studies that compared PrEP vs. No PrEP or placebo (11 studies) showed that relative risk of HIV among MSM taking PrEP was 0.24 (95% CI(0.1, 0.5)) - PrEP recipients vs. Non-PrEP recipients in open label extension showed reduced HIV incidence - Event rate of HIV among MSM highly adherent to PrEP was 0.4% (95% CI (0.1-1.0) AND 2.9% (95% CI (2.1-4.0) among moderate adherence, as compared to low adherence | PrEP use reduced risk of HIV infection  Higher adherence to PrEP further reduced risk of HIV infection |
| Experienced Homophobia and HIV Infection Risk among U.S. Gay, Bisexual, and Other Men Who Have Sex with Men: A Meta-Analysis | Jeffries 2021 | Cross-sectional (n=42), prospective (n=2)  44 studies | - Overall, among MSM experienced homophobia was associated with diagnosed HIV infection (OR=1.3, 95% CI (1.1-1.6) - In studies with >50% Black MSM, experienced homophobia was not associated diagnosed HIV infection - In studies with >50% of Latino MSM experienced homophobia was associated with diagnosed HIV infection (OR=1.7, 95% CI (1.4-2.1) - In studies with >50% of White MSM experienced homophobia was not associated with diagnosed HIV infection - No specific type of discrimination measurement (e.g., physical violence, verbal harassment, gay related stress, family mistreatment) were associated with having diagnosed HIV | Experiences of homophobia were associated with elevated chance of HIV diagnosis  Experienced homophobia increased risk of HIV infection particularly among Latino MSM |
| A meta-analysis of the efficacy of HAART on HIV transmission and its impact on sexual risk behaviours among men who have sex with men | Jiang 2020 | Cross-sectional (n=7), cohort (n=8)  18 studies | - Overall pooled incidence rate of HIV in the time of HAART is 6.63 infections / 100 per-years (95% CI (2.1-11.2)) - Per contact rate of HIV transmission in time of HAART was 0.4% (95% CI (0.2-0.6)) - One study demonstrated that HAART had a preventive effect on HIV transmission via condomless anal intercourse at the individual-level - Porco and colleagues (2004) calculated 60% decrease of the PCR of per-partnership in the post-HAART era compared with that in the pre-HAART era | Use of HAART reduced risk of HIV infection |
| Serosorting and HIV/STI Infection among HIV-Negative MSM and Transgender People: A Systematic Review and Meta-Analysis to Inform WHO Guidelines. | Kennedy 2013 | Longitudinal  4 studies | - Compared to consistent condom use, serosorting was associated with an increased odds of HIV OR=1.8, 95% CI (1.2-2.7) - Compared to no condom use, serosorting was associated with a reduced odds of HIV OR=0.5, 95% CI (0.3–0.8) - Using adjusted data did not change the relationships - Serosorting was associated with a 12% decreased risk of HIV seroconversion for every one unit increase in the natural log serosorting score (OR: 0.88; 95% CI: 0.81–0.95)   - Higher serosorting score indicated a greater likelihood of using condoms with HIV-positive or unknown partners as compared with HIV negative partners - Among HIV-negative MSM, condom use appears to be more protective against HIV and STIs than serosorting and should be encouraged | Serosorting reduced risk of HIV infection when condoms not used  Condoms were more protective against HIV infection than serosorting |
| HIV incidence among men who have sex with men in China: A meta-analysis of published studies | Li 2011 | Cohort (n=3), cross-sectional (n=9)  12 studies | - 5 studies in which association of risk factors and incident HIV infection were reported - Meta-analysis indicated that baseline syphilis infection (RR = 3.33, 95%CI, 1.97– 5.62; p,0.001), multiple sex partnership (RR = 2.81, 95%CI, 1.59–4.95; p,0.001), and unprotected receptive anal intercourse in the past six months (RR = 3.88, 95%CI, 1.44–10.47; p = 0.007) were significantly associated with HIV seroconversion | Higher numbers of sexual partners increased risk of HIV infection  Syphilis infection increased risk of HIV infection  Condomless sex was associated with increased risk of HIV infection |
| Association between rectal douching and HIV and other sexually transmitted infections among men who have sex with men: A systematic review and meta-analysis | Li 2019 | Cross-sectional (n=23), cohort (n=5)  24 studies | - Of the 20 studies reporting HIV infection, 17 found a positive association between rectal douching and HIV infection while three found no association with HIV infection - Meta-analyses revealed that MSM who reported rectal douching had a higher odds of living with HIV, OR=2.8 95% CI (2.3-3.4) - Elevated odds in cross sectional studies, as compared to cohort studies - Subgroup analyses to address confounding found similar results - Douching was not associated with increased risk of HIV infection in cohort studies (OR 1.77, 95% CI 0.9-3.7, I2 =67.1%), studies conducted in South America (OR 1.2, 95% CI 0.4-3.2, I2 =41.9%) and studies conducted in Asia (OR 2.2, 95% CI 0.9-5.5, I2 =45.5%) | Rectal douching increased risk of HIV infection in cross sectional but not cohort studies |
| HIV risk among men who have sex with men who have experienced childhood sexual abuse: Systematic review and meta-analysis | Lloyd 2012 | Cross-sectional (n=10), RCT (n=1), longitudinal (n=1)  12 studies | - Six studies indicated a greater likelihood of HIV infection among MSM with a history of childhood sexual abuse - Estimates of HIV infection among MSM with a history of childhood sexual abuse ranged from 10% to 38% - Meta-analysis indicated significantly greater odds of HIV infection among men with a history of childhood sexual abuse, as compared with men without a history OR = 1.54; 95% CI (1.22-1.95) | Childhood sexual abuse was associated with elevated risk of HIV infection |
| Risk of HIV Acquisition among Men Who Have Sex with Men Infected with Bacterial Sexually Transmitted Infections: A Systematic Review and Meta-Analysis | Malekinejad 2021 | Prospective cohort (n=18), retrospective cohort (n=6), case control (n=2)  26 studies | - Meta-analysis suggests that syphilis more than doubles HIV acquisition risk (k=21, RR 2.68, 95% CI 2.00–3.58), although with a high degree of heterogeneity (I2=66.3%, p<0.01)   - There was a greater increase in pooled RR after restricting to higher-quality data - Pooled estimate for the effect of gonorrhea on HIV risk (k=11, RR 2.38, 95% CI 1.56–3.61) with a higher degree of heterogeneity than syphilis (I2=84.2%, p<0.01)   - There was a greater increase in pooled RR after restricting to higher-quality data - Chlamydia to HIV risk pooled estimate was RR 1.99 (95% CI 1.59–2.48) with less heterogeneity (I2=30.9%, p=0.192)   - No multivariate-adjusted data were reported - Stratified meta-analysis suggests that HIV risk was similar in studies conducted in OECD-member countries (k=9, RR 2.61, 95% CI 1.44–4.74) and non-OECD countries (k=11, RR 2.52, 95% CI 1.85–3.44) | Syphilis infection elevated risk of HIV infection  Gonorrhea infection elevated risk of HIV infection  Chlamydia infection elevated risk of HIV infection |
| HIV prevalence among female sex workers, drug users and men who have sex with men in Brazil: A Systematic Review and Meta-analysis | Malta 2010 | Cross sectional (n=7), prospective cohort (n=3)  8 studies | - Among MSM focused studies, one found a four-fold increase in HIV prevalence among those men who reported unprotected anal intercourse with occasional partners (adjusted odds ratio - AOR: 3.7) - Black MSM had a three-fold greater risk of HIV, as compared to non-Black MSM (AOR: 3.4) - Another study found factors found to be independently associated with seroconversion in their sample of MSM were: "age < 25 years", "sex at the first encounter in the previous six months", and a medical history for the following infections: HBV, gonorrhea or chlamydia | Condomless anal sex with casual partners elevated risk of HIV infection  Younger age was associated with elevated HIV risk  HBV, gonorrhea, and chlamydia was associated with elevated risk of HIV infection |
| Relative Risk for HIV Infection Among Men Who Have Sex with Men Engaging in Different Roles in Anal Sex: A Systematic Review and Meta-analysis on Global Data | Meng 2015 | Cross-sectional, prospective cohort  21 studies | - Pooled incidence rates for HIV infection among men engaging in receptive anal intercourse (RAI) only, insertive anal intercourse (IAI) only and insertive & receptive anal intercourse (IRAI) were 5.0 (95 % CI 2.0–7.0), 0.8 (95 % CI 0.4–1.4) and 6.4 (95 % CI 3.2–9.6) per 100 person years, respectively - Men engaging in RAI and IRAI were 6.2 (95 % CI 3.3–11.8) and 6.6 (95 % CI 3.8–11.7) times more likely to develop incident HIV infection compared to men engaging in IAI only - Meta analyses indicated that for the latest years, 2006-2010, men engaging in RAI and IRAI, as compared to IAI, were 2.2 (95% CI 1.9-2.7) and 2.1 (95% CI 1.9-2.5) times more likely to be infected with HIV - Significant heterogeneity existed by time period of analysis, which was why subgroup analyses were conducted by 4-year increments - In 1981-85: pooled HIV prevalence among men engaging in   - Insertive anal intercourse only: 13.4 %   - receptive anal intercourse only 34.8 %   - both insertive and receptive were 35.1 % - In 1986-2010, these figures were   - Insertive anal intercourse only: 10.6 %   - receptive anal intercourse only 18.2 %   - both insertive and receptive were 19.2 % | Receptive anal sex elevated the risk of HIV infection  Insertive anal sex only decreased risk of HIV infection |
| Circumcision status and risk of HIV and sexually transmitted infections among men who have sex with men: A meta-analysis | Millett 2008 | Cross-sectional (n=19), cohort (n=2)  15 studies | - 17 studies examined the association between circumcision and HIV infection among MSM   - 9 studies found no statistically significant association with HIV   - 5 studies reported that circumcision had a significant protective association with HIV   - 1 reported a nearly significant protective association   - 1 reported that circumcised MSM had a significantly greater odds of HIV infection   - 1 reported no statistically significant association in the overall sample but found a significantly protective effect among men who only engaged in insertive anal sex - Meta analyses indicated no statistically significant association between circumcision and HIV infection even when stratified by prevalence of HIV, prevalence of circumcision, study design, geography or adjusted vs. Unadjusted analyses | Mixed relationship identified between circumcision and risk of HIV infection |
| Antiretroviral pre-exposure prophylaxis (PrEP) for preventing HIV in high-risk individuals. | Okwundu 2012 | RCT  12 studies | - The meta-analysis revealed lower HIV incidence in participants who received TDF-FTC compared to those who received placebo (Mantel-Haenszel random effects RR=0.5, 95% CI 0.3-0.9; 8918 participants) - Studies that compared TDF only versus placebo showed a significant reduction in the risk of acquiring HIV infection (RR 0.33; 95% CI 0.20 to 0.55, 4027 participants) - Among data for just MSM, the meta-analysis showed an RR=0.6, 95% CI (0.4-0.8, 1,251 participants) | PrEP use reduced risk of HIV infection |
| Human rights protections and HIV prevalence among MSM who sell sex: Cross-country comparisons from a systematic review and meta-analysis | Oldenburg 2018 | Cross-sectional  66 studies | - Fully adjusted model indicated an association between protective laws for MSM and HIV prevalence - Adjusted model showed the decrease in HIV prevalence was more than twice as large and was statistically significant: −11.0% (95% CI: −18.1%, −3.8%) - On average, countries with protective language in the penal code for MSM had 11% lower HIV prevalence compared to those without protective language - Fully adjusted model indicated an association between protective laws for sex work and HIV prevalence among men engaged in transactional sex - Adjusted model showed a decrease −7.0% (95% CI: −12.8%, −1.3%) - On average countries with protective language for sex workers in the penal code had 7% lower HIV prevalence compared to those with no protective language | Country level legal protections for MSM reduced risk of HIV infection  Country level legal protections for sex workers reduced risk of HIV infection among MSM who engage in transactional sex |
| Transactional Sex and the HIV Epidemic Among Men Who have Sex with Men (MSM): Results From a Systematic Review and Meta-analysis | Oldenburg 2015 | Cross-sectional  33 studies | - MSM who participate in transactional sex had significantly elevated HIV prevalence (OR 1.30, 95 % CI 1.08–1.57), as compared to MSM-NTS - Latin America had significantly elevated HIV prevalence among MSM-TS (OR 2.28, 95 % CI 1.87–2.78)   - When stratified by country, this elevation was noted in Ecuador, El Salvador, and Peru.   - Ecuador and Peru contained results from multivariable models assessing factors associated with HIV risk, and transactional sex was significantly associated with HIV infection in these studies. - In Sub-Saharan Africa, HIV prevalence was significantly higher among MSM-TS compared to MSM-NTS (OR 1.72, 95 % CI 1.02–2.91)   - Elevation was noted in Kenya (OR 2.56, 95 % CI 1.64–4.00) and South Africa (OR 2.88, 95 % CI 1.20–6.92)   - The association was not significant in a multivariable model in the study from South Africa - Significantly elevated HIV prevalence among men ‘‘ever’’ engaging in transactional sex compared to ‘‘never’’ (OR 1.49, 95 % CI 1.12–1.99) - In Latin America, there was a significant elevation among men who had ever engaged in transactional sex (OR 2.27, 95 % CI 1.57–3.28) - In Sub-Saharan Africa, there was a significant elevation in HIV prevalence among male sex workers compared to МSM not engaged in transactional sex (OR 2.56, 95 % CI 1.64–4.00) - In Southeast Asia, there was a significant elevation in HIV prevalence among men ‘‘ever’’ engaging in transactional sex (OR 1.56, 95 % CI 1.19–2.05) and engaging in transactional sex (OR 2.20, 95 % CI 1.38–1.52), as compared to not | Transactional sex elevated risk of HIV infection |
| Quantifying the Harms and Benefits from Serosorting Among HIV-Negative Gay and Bisexual Men: A Systematic Review and Meta-analysis | Purcell 2017 | Cross-sectional, Cohort  8 studies | - Combined effect sizes of the six studies show that serosorting, compared to condomless discordant anal intercourse is associated with a 54% reduction in HIV risk (RR = 0.46, 95% Confidence Interval [CI] 0.33–0.65) in the random-effects model - Fixed effects models showed a 48% reduction in HIV risk (RR = 0.52, 95% CI 0.45–0.61) among serosorters, as compared to condomless discordant anal intercourse - Combined effect sizes showed that serosorting, compared to no condomless anal intercourse, is associated with 64% increase in HIV risk (RR = 1.64, 95% CI 1.37–1.96) in both fixed-effect and random-effects models | Serosorting reduced risk of HIV infection when condoms were not utilized |
| Male circumcision for the prevention of human immunodeficiency virus (HIV) acquisition: a meta-analysis | Sharma 2018 | Retrospective/cross-sectional (n=34), RCT (n=3), case-control (n=3), prospective cohort (n=9)  49 studies | - Risk ratio (RR) for HIV infection among circumcised homosexual men was 0.80 (95% CI 0.69–0.92) - Circumcision was therefore protective for both sexualities, but there was a greater benefit for heterosexual men - Circumcision was not definitively found to have a protective effect amongst predominantly insertive homosexual men (RR: 0.44, 95% CI 0.17–1.18) nor receptive men (RR: 0.83, 95% CI 0.63–1.09)--but sample sizes were small | Circumcision not significantly associated with HIV risk among MSM |
| HIV testing and engagement with the HIV treatment cascade among men who have sex with men in Africa: a systematic review and meta-analysis | Stannah 2018 | Cross-sectional (n=64), cohort (n=10)  75 studies | - Univariate meta-regression indicated a higher proportion of HIV positive MSM aware of their status was associated with   - Not living in eastern Africa (p=0·046),   - Less repressive legislation (p=0·014),   - Less severe penalties for same-sex relations (p=0·00023), and   - Lower global anti-LGBT legislation index score (p=0·0050) - For these three outcomes (ever tested, tested in the past 12 months, and HIV-positive aware of their status), proportions were 57·4%, 35·5%, and 6·7%, respectively, for countries with the most severe legislation compared with 71·6%, 49·3%, and 22·0%, respectively, for countries with the least severe legislation p<0.005 | Less repressive same-sex policies were associated with elevated knowledge about HIV status  Less awareness of HIV-positive status in countries with more discriminatory same-sex policies |
| A systematic review of evidence to inform HIV prevention interventions among men who have sex with men in Europe | Stromdahl 2015 | RCT, Cohort  24 studies | - Condom use, universal ARV coverage, TaSP, & peer outreach interventions had a high HASTE grade for HIV prevention - Consistent efficacy data showed that condom use during anal intercourse prevents HIV transmission RR=0.36; 95% CI 0.20–0.67 - A randomised, double-blinded controlled trial with 1,763 serodiscordant heterosexual couples and 37 serodiscordant male MSM couples, reported a relative reduction of 96% in the number of linked HIV-1 transmissions cases resulting from the early initiation of antiretroviral therapy, as compared with delayed therapy - PrEP research indicates a 44% reduction in the incidence of HIV (95% CI 15–63).   - Detectable FTC–TDF blood levels strongly correlated with the prophylactic effect - Among men having primarily insertive anal intercourse there was a significant decrease in HIV infection (OR: 0.27; 95% CI 0.17–0.44) - A comprehensive review of 21 observational studies with a total of 71,693 participants found **insufficient evidence** that male circumcision prevents acquisition of HIV - **HASTE 3**: systematic review including three observational studies found that serosorting increased HIV transmission by 79% compared with condom use; compared with no condom use, serosorting reduced HIV transmission by 53% | Condom use reduced risk of HIV infection  Early initiation of ARVs reduced risk of HIV infection  PrEP use reduced risk of HIV infection  Insertive sex reduced risk of HIV infection among circumcised MSM  Insufficient evidence that circumcision reduces HIV risk  Serosorting reduced risk of HIV infection when condoms not used  Peer outreach may reduce HIV risk |
| Amphetamine-type stimulants and HIV infection among men who have sex with men: Implications on HIV research and prevention from a systematic review and meta-analysis | Vu 2015 | Cross-sectional, case control, longitudinal  35 studies | - Association between amphetamine type stimulants (ATS) and HIV infection was significant in all study designs - In cross-sectional studies, MSM who reported ever using amphetamine type stimulants (ATs) were 1.70 times more likely to be infected with HIV than non-users (Prevalence rate ratio (PRR)=1.70; 95% CI: 1.47-1.98) - In case-control studies, the pooled OR=2.90 (95% CI: 2.04- 4.12) - In longitudinal studies, the pooled hazards ratio (HR)=3.13 (95% CI: 2.65-3.70) - In the meth/amphetamine subgroup, the pooled estimate was statistically significant in all study designs   - PRR for cross-sectional studies was 1.85; 95% CI: 1.57-2.17   - OR for case-control studies was 2.73; 95% CI: 2.16-3.46   - HR for longitudinal studies was 3.43; 95% CI: 2.98-3.95 - In the ecstasy subgroup there were mixed results   - In cross-sectional studies, the pooled PR estimate was not statistically significant (PR1.15; 95% CI: 0.88-1.49)   - In case-control studies, the pooled OR estimate was significant (OR3.04 (95% CI: 1.29-7.18)   - In longitudinal studies, the pooled HR estimate was statistically significant (HR2.48; 95% CI: 1.42-4.35) | Use of amphetamine drugs associated with increased risk of HIV infection  Use of ecstasy associated with increased risk of HIV in cohort studies |
| Sexual Risk Behaviors and HIV Infection among Men Who Have Sex with Men and Women in China: Evidence from a Systematic Review and Meta-Analysis | Wang 2015 | Cross-sectional  36 studies | - Across studies, a higher prevalence of HIV was found among MSM who have sex with women too, as compared with MSM only (6.6% versus 5.4%, OR = 1.27, 95% CI = 1.01–1.58) - Subgroup analysis by region showed variation in HIV prevalence between MSMW and MSMO - For studies that collected data in 2009 and later, the gap in HIV prevalence between MSMW and MSMO was larger (8.3% versus 5.6%, OR = 1.59, 95% CI = 1.08–2.33) | Mixed gender sexual relationships may increase risk of HIV infection |
| The use of geosocial networking smartphone applications and the risk of sexually transmitted infections among men who have sex with men: a systematic review and meta-analysis. | Wang 2018 | Cross-sectional  30 studies | - Pooled random effects OR=0.89 (95% CI, 0.68–1.16) for HIV diagnosis, which indicates no significant difference in HIV infection between app-users and non-users | App usage did not increase risk of HIV infection |
| HIV Nonoccupational Postexposure Prophylaxis among Men Who Have Sex with Men: A Systematic Review and Meta-Analysis of Global Data | Wang 2020 | Cross sectional (n=36), cohort (n=14), intervention (n=7)  74 studies | - Fourteen studies reported a total of 500 HIV seroconversions among 19,546 MSM who had been prescribed nPEP - Nine of the 14 studies reported HIV incidence in MSM after nPEP uptake (range: 1.0–7.6 per 100 person-years, median: 2.2 per 100 person years) - Six of eight studies that reported the interval between nPEP initiation and HIV diagnosis indicated that the majority of seroconversions tested HIV-negative at >3 months post-nPEP uptake, implying that these seroconversions were unlikely due to nPEP failure. | PEP reduced risk of HIV infection |
| HIV and Viral Hepatitis among Imprisoned Key Populations | Wirtz 2018 | Cross-sectional  43 studies | - Among populations imprisoned, the prevalence rate ratio (PPR) of HIV among MSM was 5 times (pooled PPR = 5.3, 95% CI: 3.5, 7.9; P < 0.001) than that of their male counterparts who did not report having sex with men - Prevalence of HIV was more than 10 times higher among MSM than that of male prisoners who did not report having sex with men in the Latin America and Caribbean region - HIV prevalence was almost 20 times higher among MSM compared with their male counterparts in the Western Europe region | MSM who experience incarceration were at elevated risk of HIV infection |
| Male circumcision for prevention of homosexual acquisition of HIV in men. | Wiysonge 2011 | Cohort (6), case-control (1), cross-sectional (14)  21 studies | - Main analysis indicated no statistically significant association between male circumcision status and HIV infection in men who have sex with men (Analysis 1.1: 20 studies with 65,784 participants; OR 0.86, 95% CI 0.70 to 1.06)   - Inconsistency in study results seemed to be explained by sexual roles/positions - Statistically significant decrease in HIV infection found in studies of circumcised men who reported a predominantly or exclusively insertive role during anal sex: 7 studies with 3465 participants OR 0.27, 95% CI 0.17 to 0.44; I2 = 0% - No statistically significant association found between male circumcision and HIV infection in studies of men who reported a mainly receptive role during anal sex (3 studies with 1792 participants; OR 1.20, 95% CI 0.63 to 2.29; I2 = 0%), and in studies where differentiation between men who reported insertive versus receptive role was not clear or not done (17 studies, 64,538 participants; OR 1.00, 95% CI 0.92 to 1.09; (P = 0.90); I2 = 0%) | Circumcision was not associated with reduction in HIV risk  Lower risk of HIV infection associated with insertive anal sex  Associations are likely due to positioning rather than circumcision status  No significant association found between male circumcision and HIV infection among men participating in receptive anal sex |
| Identifying Resilience Resources for HIV Prevention Among Sexual Minority Men: A Systematic Review | Woodward 2017 | N/A  20 studies | - One study in the review indicated that serosorting reduced HIV seroconversion (p<0.000) | Serosorting reduces risk of HIV infection |
| Effect of syphilis infection on HIV acquisition: A systematic review and meta-analysis | Wu 2021 | Cohort, Case-Control  22 studies | - Random-effects meta-analysis indicated the HIV incidence was significantly higher among patients with syphilis infection as compared to the syphilis-negative control group, especially in the incident syphilis infection group (RR 3.21, 95%CI 2.26 to 4.57; n=5, I2 =0%) - The pooled RR was 2.67 (95% CI 2.05 to 3.47; n=17, I2 =45.5%) for exposure to prevalent syphilis, as compared to negative - The results and the magnitude of the associations were similar among MSM and for other high-risk populations (RR 2.60, 95% CI 1.78 to 3.80; n=12; p<0.05) vs. (RR 2.98, 95%CI 2.15 to 4.14; n=5, respectively) | Syphilis infection associated with elevated risk of HIV infection |
| Circumcision to prevent HIV and other sexually transmitted infections in men who have sex with men: a systematic review and meta-analysis of global data | Yuan 2019 | Cross-sectional (n-45), cohort (n=15), case-control (n=2)  62 studies | - Of the 45 studies that examined the association between circumcision and HIV status among MSM, 29 reported non-significant associations. - Eleven studies found circumcision to have a statistically significant protective association with HIV infection among all MSM. - Two studies found a significant protective association with circumcision only among MSM who primarily engage in insertive anal sex - Two studies reported a significant protective association with circumcision only among men who have sex with both men and women (MSMW) - One included studies that found circumcised MSM to be at significantly increased odds of HIV infection - Overall meta-analyses indicted that circumcision was associated with 23% lower odds of HIV infection in MSM overall (OR 0·77, 95% CI 0·67-0·89; number of estimates [k]=45; I 2=77%)   - The cumulative meta-analysis suggested that this protective association became evident since 2011 - MSM in low & middle income countries saw a more protective association (95% CIs did not overlap) (0·58, 0·41-0·83; k=23; I 2=77%), as compared to high-income countries (0·99, 0·90-1·09; k=20; I 2=40%) - Compared to the overall pooled estimate, this protective association remained significant and tended to be stronger among MSM from Southeast Asia or Africa, MSM who primarily engage in insertive anal sex, younger MSM, non-clinic-based studies, and studies in which the proportion of MSM self-reporting regular condom use was lower - Restricting the meta-analysis to the 13 studies that adjusted for confounders elevated the magnitude of the protective association between circumcision and HIV infection (OR 0·64, 95% CI 0·45-0·93; k=15; I 2=87%) | Circumcision has mixed results for reducing risk of HIV infection  Circumcision was more protective in LMIC compared to high income countries |
| Voluntary medical male circumcision and HIV infection among men who have sex with men: Implications from a systematic review | Zhang 2019 | Cross-sectional (n=24), cohort (n=9)  37 studies | - Among 33 studies, four studies revealed that circumcision significantly reduced the odds of HIV infection among MSM, while 29 studies reported that circumcision had no statistically significant associations with HIV - Overall effect size of circumcision on HIV infection was statistically significant (aOR, 0.93; 95% CI, 0.88–0.99) - Sugroup analyses by sexual position indicated that after removing 1 outlier study, the odds of HIV infection among circumcised MSM who primarily/exclusively practiced insertive sex suggested a protective effect against HIV (aOR, 0.51; 95% CI, 0.23–1.11) - Among all 33 studies, 24 employed a cross sectional study design and a protective and significant association was found among these studies (OR, 0.92; 95% CI, 0.87–0.98) - Nine studies with a cohort design revealed a non-significant association (aOR, 1.01; 95% CI, 0.86–1.19) - The evidence for the potential protective effect of voluntary medical male circumcision was stronger among men who have sex with men in Asia and Africa   - Among non-Asian MSM, there was no protection of circumcision against HIV infection (aOR, 0.97; 95% CI, 0.91–1.03). (b)   - Among studies conducted in Asian countries, the odds of being HIV infected was 31% lower compared with the odds of HIV infection among uncircumcised (aOR, 0.69; 95% CI; 0.58–0.81) | Circumcision not associated with reduction in HIV risk  Insertive anal sex may be a protective factor against HIV  Protective effect of circumcision was stronger among MSM in Asia and Africa |
| HIV incidence and associated risk factors in men who have sex with men in Mainland China: An updated systematic review and meta-analysis | Zhang 2016 | Prospective cohort  25 studies | - Factors associated with the HIV seroconversion rate of MSM were obtained through subgroup analysis of 10 articles - Factors associated with HIV seroconversion were:   - Age <25 years (v. 25 years; RR = 1.85, 95% CI: 1.29–2.65, P < 0.01)   - Having below junior college education (v. junior college or higher education; RR = 1.87, 95% CI: 1.46–2.49, P < 0.01),   - 2 male partners in the past 6 months (RR = 2.50, 95% CI: 1.75–3.56, P < 0.01),   - Baseline syphilis infection (RR = 2.99, 95% CI: 2.17–4.13, P < 0.01),   - Self-identified homosexual orientation (v. other sexual orientations; RR = 1.91, 95% CI: 1.10–3.33, P < 0.01),   - Preferred bottom or versatile roles in anal sexual intercourse (RR = 2.33, 95% CI: 1.29–4.23, P < 0.01), and   - Unprotected anal intercourse in the past 6 months (RR = 2.16, 95% CI: 1.18–3.97, P = 0.01) were statistically significantly associated with an HIV seroconversion event - Uncircumcised MSM had a marginally significantly higher HIV incidence than circumcised MSM (RR = 3.35, 95% CI: 0.99–11.23, P = 0.051) | Various factors associated with increased risk of HIV infection such as younger age, less education, more sexual partners, syphilis infection, receptive anal sex, and condomless sex |
| Association of nitrite inhalants use and unprotected anal intercourse and HIV/syphilis infection among MSM in China: A systematic review and meta-analysis | Zhang 2020 | Cross-sectional  15 studies | - Authors used data from the 9 studies that utilized laboratory-based testing to compare HIV rates among nitrite inhalants users and nonusers - Pooled prevalence of HIV was 15.4% [469/3047] vs. 9.4% [1001/10664] (OR = 1.83 [95% CI: 1.29–2.60]; I 2 = 85%; P < 0.01) among nitrite inhalants users than nonusers, respectively | Nitrites use elevated risk of HIV infection |
| Characteristics of Men Who Have Sex With Men Who Use Smartphone Geosocial Networking Applications and Implications for HIV Interventions: A Systematic Review and Meta-Analysis | Zou 2017 | Cross-sectional  17 studies | - Meta-analysis showed that app-using MSM were less likely to have HIV infection (OR=0.63, 95 %CI: 0.4–0.95), as compared to non-app-using MSM - Compared to non-app-using MSM, app-using MSM were more likely to have tested for HIV in lifetime (Pooled odds ratio = 2.1, 95 % confidence interval: 1.7–2.6) and have similar HIV prevalence | App-using MSM did not have elevated risk of HIV infection |
